# Suppression of m6A mRNA modification by DNA hypermethylated *ALKBH5* aggravates the oncological behavior of KRAS mutation/LKB1 loss lung cancer

**DOI:** 10.1038/s41419-021-03793-7

**Published:** 2021-05-20

**Authors:** Donghong Zhang, Jinfeng Ning, Imoh Okon, Xiaoxu Zheng, Ganesh Satyanarayana, Ping Song, Shidong Xu, Ming-Hui Zou

**Affiliations:** 1 Center for Molecular and Translational Medicine, Georgia State University, 157 Decatur St SE, Atlanta, GA 30303 USA; 2 The Thoracic Department of Harbin Medical University Cancer Hospital, 150 Haping Road, Harbin, 150040 China

**Keywords:** Cancer epigenetics, Non-small-cell lung cancer

## Abstract

Oncogenic *KRAS* mutations combined with the loss of the LKB1 tumor-suppressor gene (KL) are strongly associated with aggressive forms of lung cancer. *N*6-methyladenosine (m6A) in mRNA is a crucial epigenetic modification that controls cancer self-renewal and progression. However, the regulation and role of m6A modification in this cancer are unclear. We found that decreased m6A levels correlated with the disease progression and poor survival for KL patients. The correlation was mediated by a special increase in ALKBH5 (AlkB family member 5) levels, an m6A demethylase. *ALKBH5* gain- or loss-of function could effectively reverse LKB1 regulated cell proliferation, colony formation, and migration of KRAS-mutated lung cancer cells. Mechanistically, LKB1 loss upregulated ALKBH5 expression by DNA hypermethylation of the CTCF-binding motif on the *ALKBH5* promoter, which inhibited CTCF binding but enhanced histone modifications, including H3K4me3, H3K9ac, and H3K27ac. This effect could successfully be rescued by LKB1 expression. *ALKBH5* demethylation of m6A stabilized oncogenic drivers, such as *SOX2, SMAD7*, and *MYC*, through a pathway dependent on YTHDF2, an m6A reader protein. The above findings were confirmed in clinical KRAS-mutated lung cancer patients. We conclude that loss of LKB1 promotes *ALKBH5* transcription by a DNA methylation mechanism, reduces m6A modification, and increases the stability of m6A target oncogenes, thus contributing to aggressive phenotypes of *KRAS-*mutated lung cancer.

## Introduction

Recent global cancer statistics confirm that lung cancer is a commonly diagnosed malignancy and a leading cause of cancer-related deaths in both men and women^1^. Non-small cell lung cancer (NSCLC) accounts for about 85% of all lung cancers, and is not sensitive to most available treatment options. Approximately 10% of NSCLC cases involve concurrent *KRAS* mutation and LKB1 loss (KL)^2–4^. These cases are often more aggressive in terms of metastatic spread and drug resistance^5–9^ than those with neither mutation nor loss. However, the underlying molecular basis of these aggressive clinical behaviors is obscure.

Epigenetic modifications, including DNA/RNA methylation and histone methylation/ acetylation, have important roles in carcinogenesis^10^. KL cancer cells have higher levels of *S*-adenylmethionine (SAM) synthesis and DNA methylation^11^. SAM increases substrate supply to DNA and histone enzymes, such as DNMT1 and EZH2. Elevated SAM levels may also fuel RNA methylation^12^, suggesting that KL-mutations mediate a distinct form of epigenetic dysregulation. Nevertheless, reports are lacking on the regulation and role of RNA methylation in KL cancer cells.

*N*6-methyladenosine (m6A) mainly occurs at the consensus motif of GGm6ACC, and is the most prevalent internal chemical modification of mRNAs in eukaryotes^13^. Functionally, the reversible m6A modification of mRNAs is critical to cancer self-renewal and malignancy of several tumors^14–17^, including glioblastoma^18^, acute myeloid leukemia^19^, hepatocellular carcinoma^20^, and breast cancer^21^. In this regard, investigation of the landscapes and functions of m6A modification is an emerging research frontier known as RNA epigenetics or epitranscriptomics. AlkB homolog 5 (ALKBH5), a de-methyltransferase of m6A, is more highly expressed in most tissues than other m6A modulators^8^. ALKBH5 deficiency leads to compromised spermatogenesis in mice, and displays widespread mRNA methylation and global RNA instability^22^. Most recently, ALKBH5 was found to have oncogenic roles in glioblastoma and breast cancer cells^17,20^, suggesting it contributes to mRNA m6A methylation in cancer.

This study investigated the role and underlying mechanism of m6A modulation in aggressive KL mutant lung cancer cells. We found that loss of LKB1 activity promotes ALKBH5 transcription by a DNA methylation dependent mechanism. ALKBH5 demethylated and stabilized m6A modification target oncogenes in KRAS mutant cancers, which may influence how vulnerable the cancer is to therapy.

## Results

### Reduced m6A level was associated with aggressive KL lung cancer

**Fig. 1:**
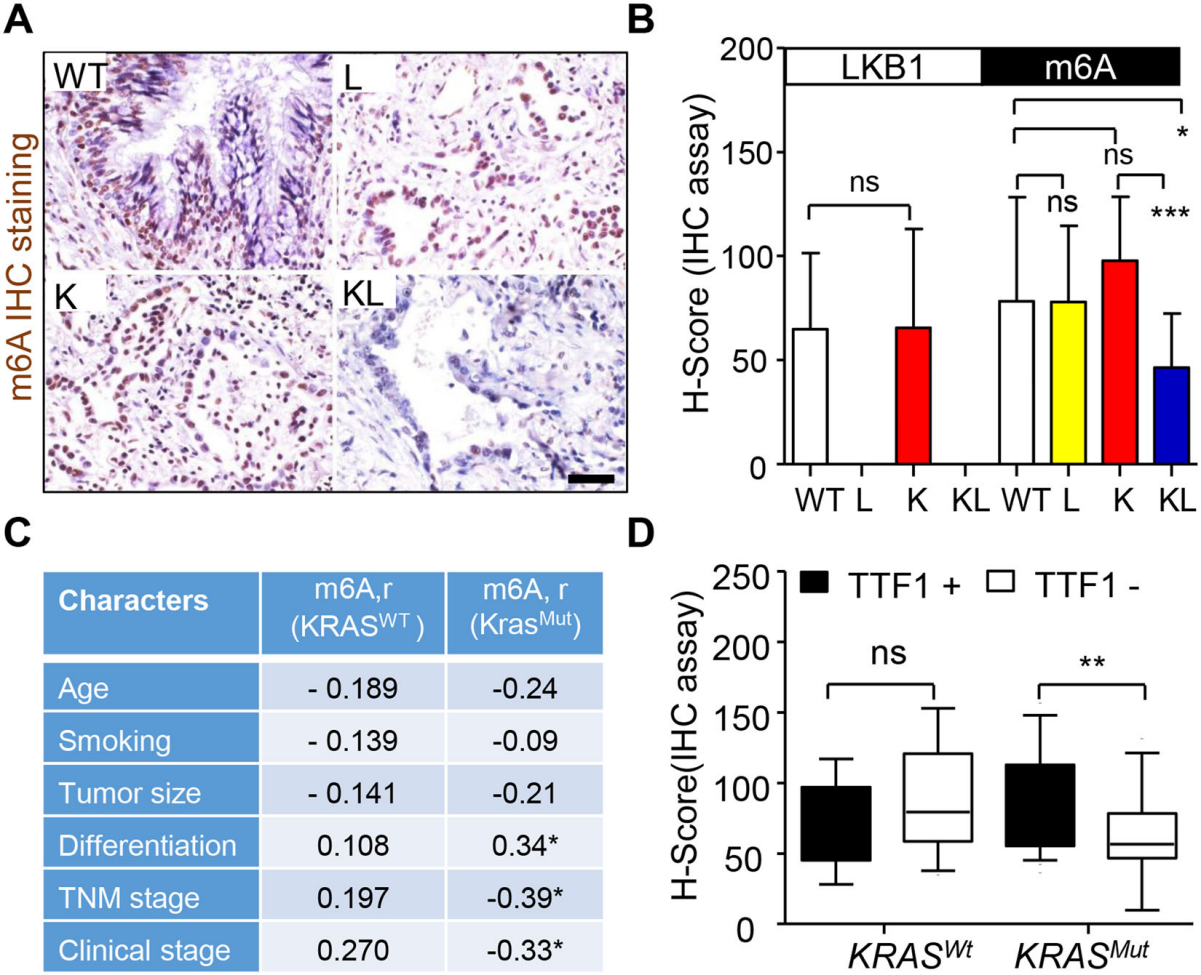
Reduced m6A modification is associated with the aggressiveness of lung cancer with *KRAS* mutation and LKB1 loss. **A**, **B** Representation and quantification of LKB1 expression and m6A level in lung cancer patients. Data were mean ± SD. Bar = 50 µm. **C** Spearman correlation analysis of m6A level with the clinical characters of KRAS mutant or wild-type lung cancer patients. **D** The m6A level in lung cancer patients with TTF1 positive or negative. Boxes and whiskers represent the 10th to 90th percentiles, respectively; the median is the central line in each box. **P < 0.01, ***P < 0.001 by Student’s *t*-test. *KRAS* ^Mut^; LKB1 ^Loss^ (KL); *KRAS* ^Mut^; LKB1 ^Wt^ (K); *KRAS* ^Wt^; LKB1 ^Loss^ (L) and *KRAS* ^Wt^; LKB1 ^Wt^ (WT).

We first investigated the association between m6A RNA modification and aggressive KL lung cancer. IHC staining showed the reduced m6A level by loss of LKB1 in patients with *KRAS* mutation. And, the lowest of m6A level was found in KL tumor tissues when compared to wild type (WT), *KRAS* ^Wt^; LKB1 ^Loss^ (L), or *KRAS* ^Mut^; LKB1 ^Wt^ (K) tumor tissues (Fig. 1A, B). The m6A level was inversely correlated with TNM (Tumor, Node, and Metastasis) and clinical stage, whereas positively with tumor differentiation in *KRAS* mutant patients, but not in those with Kras wildtype (Fig. 1C). In addition, we found lower m6A level in K specimens that were null for thyroid transcription factor 1 (TTF1), a positive prognostic feature (Fig. 1D)^23^. Thus, reduced m6A modification is associated with aggressive KL lung cancer.

### Loss of LKB1 enhanced ALKBH5 responses for m6A reduction in K lung cancer

**Fig. 2:**
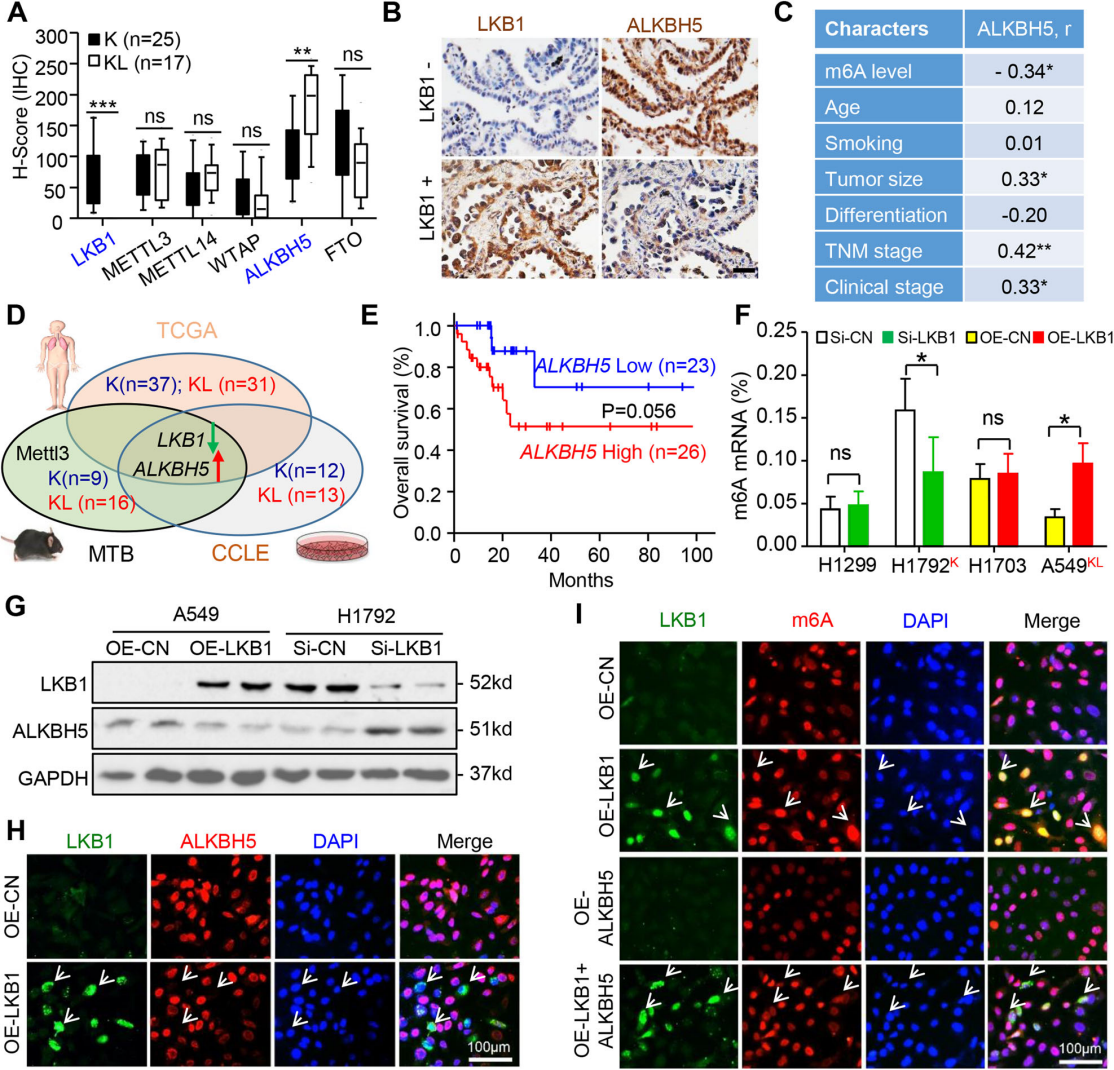
LKB1 loss upregulated ALKBH5 responses to m6A reduction in KRAS mutant lung cancer. **A**, **B** Quantification and representation of LKB1 and m6A modulators in KL relative to K lung cancer patients. Boxes and whiskers represent the 10th to 90th percentiles, respectively; the median is the central line in each box. Bar = 50 µm. **C** Spearman correlation analysis of ALKBH5 expression with the KRAS mutant lung cancer pathological characters. **D** Venn diagram showing the differentially expressed genes (DEGs) of *LKB1*, m6A modulators, and readers between KL and K lung cancer. **E** Kaplan–Meier survival curve of patients with high and low *ALKBH5* mRNA expression from the TCGA dataset. **F** Global m6A level regulation by *LKB1* in lung cancer cell lines. **G** Western blot showing the LKB1 and ALKBH5 protein expression. **H** Co-immunofluorescence staining for LKB1 and ALKBH5 in A549 cells. Arrows show LKB1 positive and ALKBH5 negative cells. **I** LKB1 and m6A co-staining by LKB1 and/or ALKBH5 overexpression in A549 cells. Arrows show cells of LKB1 positive and m6A with strong (Second panel) or weak (Forth panel) intensity. Data were mean ± SD in **F** (*n* = 5). *P < 0.05 (Student’s *t*-test). Si-CN, SiRNA-A; OE-CN, OE-c-Flag pcDNA3; OE, overexpression. *KRAS* ^Mut^; LKB1 ^Loss^ (KL); *KRAS* ^Mut^; LKB1 ^Wt^ (K).

To investigate how m6A is regulated in KL lung cancer, we compared the expression of proteins that act as the m6A writer complex (*METTL3, METTL14*, and *WTAP*) and erasers (*ALKBH5* and *FTO*). IHC staining showed that only ALKBH5 expression was higher in KL than that of in K tumor tissues (Fig. 2A, B). ALKBH5 protein expression was negatively correlated with m6A level, in contrast to positive correlation with the tumor size, TNM and clinical stage in K lung cancer patients (Fig. 2C). However, LKB1 loss alone was not sufficient to change m6A level or ALKBH5 expression or their relationship with aggressive tumor phenotypes (Fig. S1 A–D).

To confirm observations of clinical specimens, we screened for the differential expressions of m6A mediators and readers based on database queries. Analysis of TCGA, MTB^8,24^, and CCLE databases revealed that *ALKBH5* mRNA expression was consistently higher in KL cancer tissues or cells, and negatively correlated with *LKB1* expression (Figs. 2D and S2A–D). Furthermore, elevated ALKBH5 expression correlated with poor prognosis for patients with *KRAS* mutation (Fig. 2E). Additionally, ALKBH5 had the highest basal mRNA expression in human and mouse lung cancer tissues and cell lines (Fig. S2A–C). Taken together, these findings indicate that *ALKBH5*, a major regulator of m6A modification, contributes to aggressive phenotypes of KL lung tumors.

To investigate whether LKB1 loss affects ALKBH5 and m6A modification, we screened several lung cancer cell lines and categorized them based on KRAS mutation and LKB1 expression status. A549^KL^ (*KRAS*^*mut*^/*LKB1*^*loss*^), H1792^K^ (*KRAS*^*mut*^/*LKB1*^*high*^), H1299 (*KRAS*^*wt*^/*LKB1*^*high*^), and H1703 (*KRAS*^*wt*^/*LKB1*^*low*^) were selected from each category for further analyses (Fig. S3A). We then generated the loss of LKB1 function by siRNA-LKB1-transfection in H1299 and H1792 cells, while gain-of-function by *LKB1* overexpression in H1703 and A549 cells. As expected, LKB1 expression positively regulated m6A levels in cells with *KRAS* mutation, but not with *KRAS* wild type (Fig. 2F). Consistent with clinical data, western blot and qRT-PCR assays showed that *ALKBH5* was also the only m6A modulator negatively regulated by LKB1 in *KRAS*^*mut*^ cells, but was unaffected in *KRAS*^*wt*^ cells (Figs. 2G and S3B–E). These observations were supported by immunofluorescence staining that demonstrated negligible *ALKBH5* signal in LKB1 overexpression A549 cells (Fig. 2H). Exogenous ALKBH5 expression blocked m6A staining in the presence of LKB1 (Fig. 2I). Furthermore, LKB1 expression negatively correlated with ALKBH5 expression was also found in *KRAS*-mutated pancreatic and colorectal cancer cell lines (Fig. S4A–E). Therefore, *LKB1* expression directly affects the global m6A levels via *ALKBH5* in KRAS-mutated cancer cells.

### *ALKBH5* negatively regulated *LKB1* role for lung cancer cell proliferation and migration

**Fig. 3:**
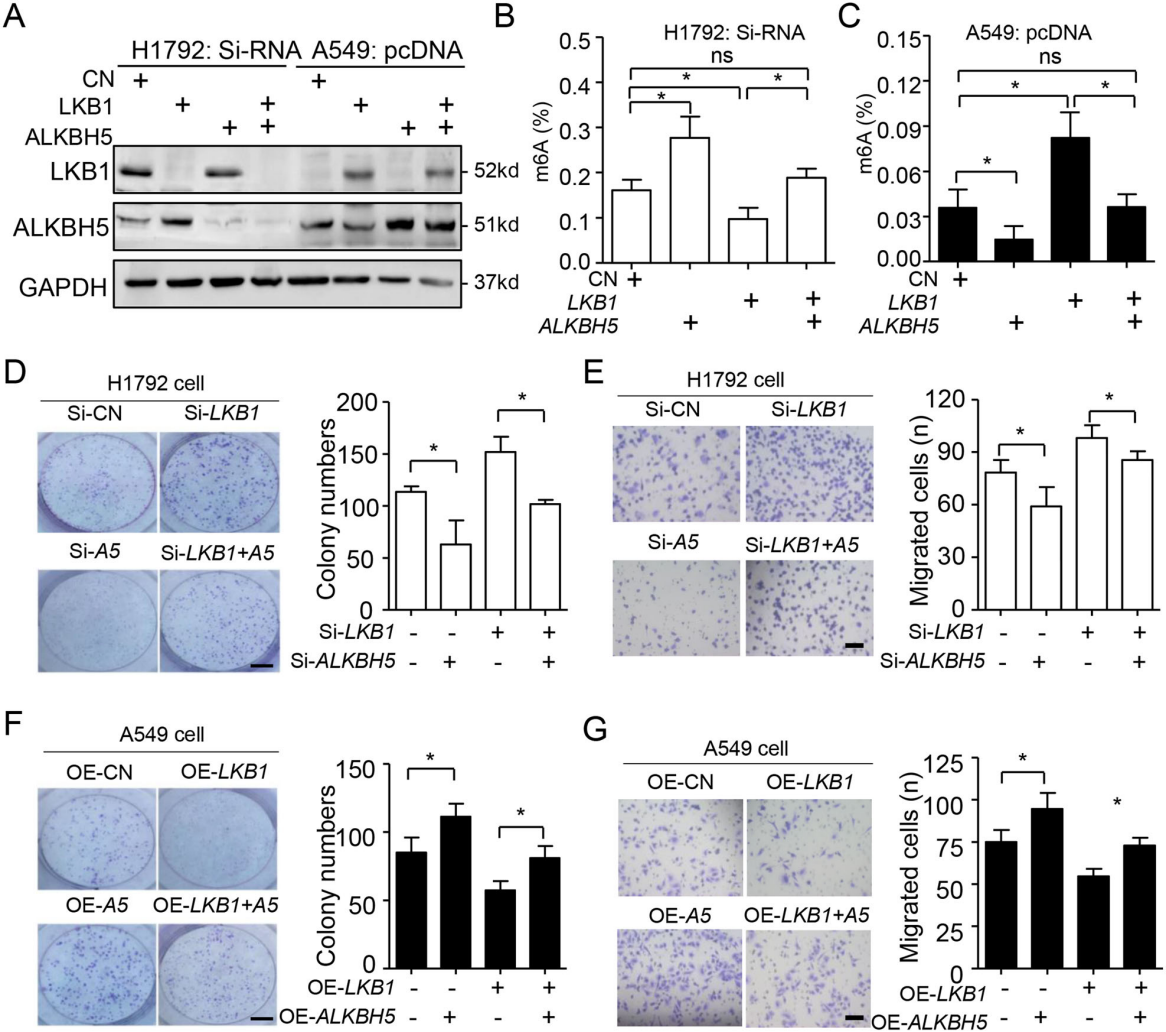
ALKBH5 increases the lung cancer cell proliferation and migration. **A** Western blot analysis shown that LKB1 and ALKBH5 were successfully knocked down in H1792 cells and overexpressed in A549 cells. **B**, **C** The measurement of global m6A by ELISA assay in H1792 (**B**) or A549 (**C**) cells, respectively. The colony formation ability (**D**) and cell migration (**E**) were reduced at both of basal level and LKB1 silenced level by ALKBH5 knockdown in H1792 cells, respectively. The reversible effect was found at both of basal level and LKB1 overexpressed level by ALKBH5 transfected in A549 cells using colony formation (**F**) and transwell assay (**G**). Data were mean ± SD (*n* = 5) and were analyzed by one-way ANOVA, followed by Bonferroni’s multiple comparison test for **B**–**G**. *P < 0.05. Bar = 5 mm in **D** and **F**; Bar = 50 µm in **E** and **G**.

Next, we explored functional relationships between LKB1 and ALKBH5 in *KRAS* mutant lung cancer cells. First, we established the transient *ALKBH5* and/or *LKB1* knockdown models in H1792 cells, as well as overexpression in A549 cells (Fig. 3A). As expected, *ALKBH5* could fully release *LKB1* repressed m6A levels in both cell lines (Fig. 3B, C). Functionally, *ALKBH5* knockdown significantly inhibited cell proliferation, as shown by decreased colony formation in *LKB1*-silenced H1792 cells (Fig. 3D). We observed similar for H1792 cell migration in the transwell assay (Fig. 3E). Conversely, *ALKBH5* overexpression promoted cell proliferation and migration in *LKB1*-transfected A549 cells (Fig. 3F, G). Thus, *ALKBH5* negatively regulated *LKB1* role for cell proliferation and migration in KRAS mutant cells.

### Loss of *LKB1* upregulated *ALKBH5* via DNA hypermethylation in *KRAS* mutant cancer cells

**Fig. 4:**
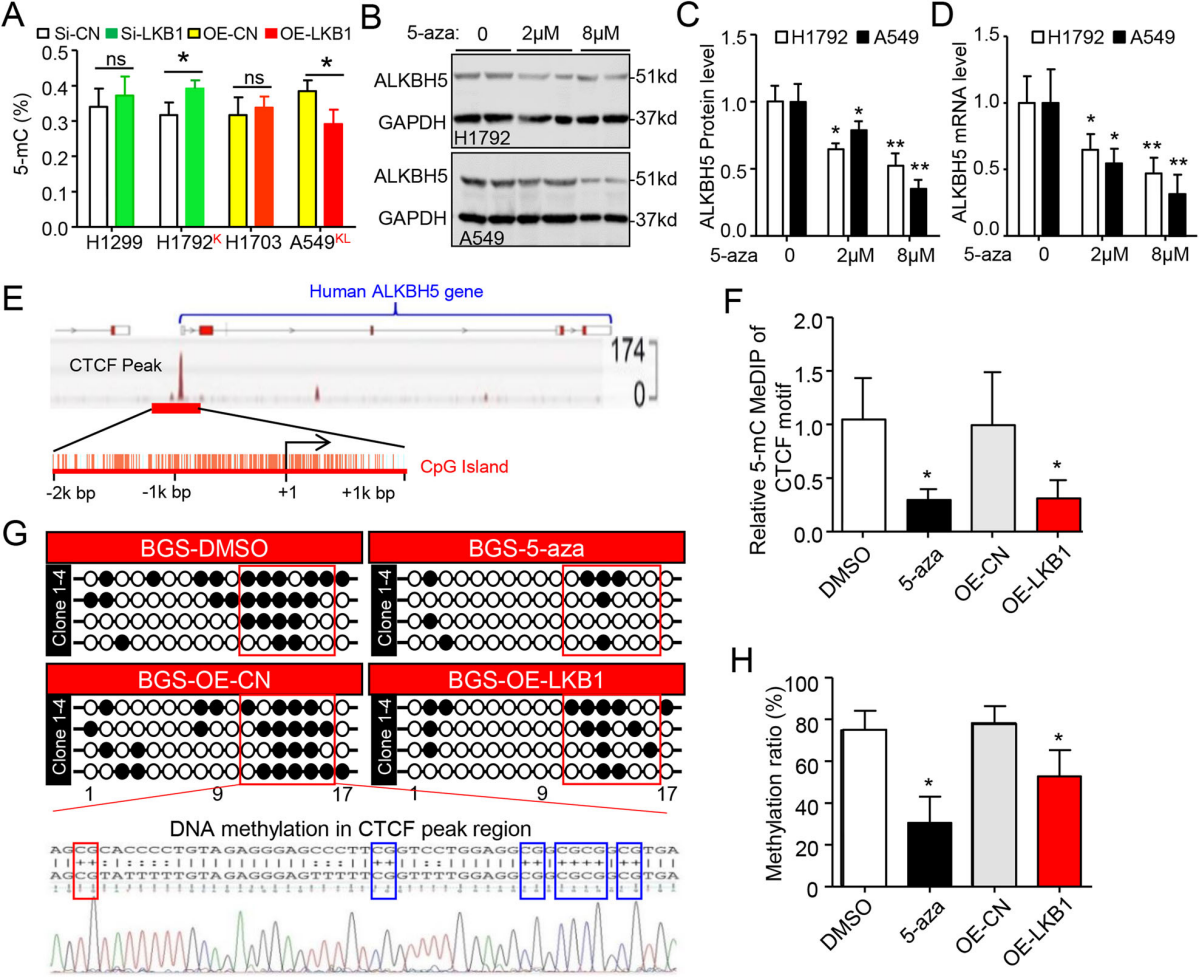
DNA hypermethylation upregulates *ALKBH5* in KL lung cancer cells. **A** 5mC DNA methylation was detected by ELISA assay. Data as mean ± SD (*n* = 5), *P < 0.05 (Student’s *t*-test). **B**–**D** Representation and quantification of ALKBH5 expression at protein (**B**, **C**) and mRNA levels (**D**) in response to 5-aza treatment in A549 and H1792 cells for 72 h. Error bars, SD (*n* = 4 for WB; *n* = 5 for qRT-PCR), *P < 0.05 as compared their corresponding controls (2-way ANOVA with Bonferroni multiple comparison post hoc test). **E** CTCF peak located in the CpGs Island of human *ALKBH5* gene promoter. Bottom panel: the Methprimer histogram of CpG islands (CpGI, red box) and CpG dinucleotides (red vertical lines) in the regulatory region of *ALKBH5*. **F** MeDIP assay showing the decrease of 5mC enrichment on the *ALKBH5* promoter containing CTCF motif fragment by treated with 5-aza and LKB1 overexpression. **G**, **H** Representative bisulfite sequencing of four clones (**G**) and quantification of DNA methylation (**H**) of *ALKBH5* promoter containing CTCF motif. *n* = 4 in **F** and **H**, mean ± SD, * *P* < 0.05 vs. A549 DMSO by one-way ANOVA followed by Tukey’s test in **F** and **H**. Si-CN, Si-RNA-A. OE-CN, OE-c-Flag pcDNA3. OE overexpression.

We then explored how *LKB1* regulates *ALKBH5* through DNA methylation. Consistent with previous studies in pancreatic ductal epithelial cells^11^, *LKB1* alterations also negatively regulated the global 5mC DNA methylation in *KRAS* mutant lung cancer cells (Fig. 4A) but had no effect in *KRAS* wild-type cells. Specifically, *ALKBH5* gene DNA methylation positively correlated with *ALKBH5* mRNA expression in *KRAS* mutant lung, and colorectal cancer cell lines, based on the CLLE database (Fig. S5A–D). Subsequent treatment with 5-azacytidine (5-aza), an inhibitor of DNA methylation, reduced ALKBH5 mRNA and protein expression in a dose-dependent manner in H1792 and A549 cells, independent of LKB1 status (Fig. 4B–D). Based on ChIP-sequence analysis from the ENCODE database, we identified a single putative transcriptional repressor, CTCF (CCCTC-binding factor) and several activators, including H3K4me1, H3K4me2, H3K4me3, H3K9ac, and H3K27ac, on the ALKBH5 gene core promoter in A549 cells (Fig. S6). Interestingly, the CTCF peak region was colocated in the CpG island of ALKBH5 promoter (Fig. 4E). Further analysis by MeDIP assay indicated 5mC-methylation on the CTCF peak region was decreased with 5-aza treatment or exogenous *LKB1*-transfection in A549 cells (Fig. 4F). Lastly, bisulfite sequencing confirmed decreased DNA methylation on the CTCF peak region in 5-aza-treated or *LKB1* overexpressed A549 cells (Fig. 4G, H). Thus, the data indicate that LKB1 loss induces DNA hypermethylation, thereby controlling *ALKBH5* expression in *KRAS* mutant cancer cells.

### DNA hypermethylation of the CTCF motif is critical for ALKBH5 upregulation

**Fig. 5:**
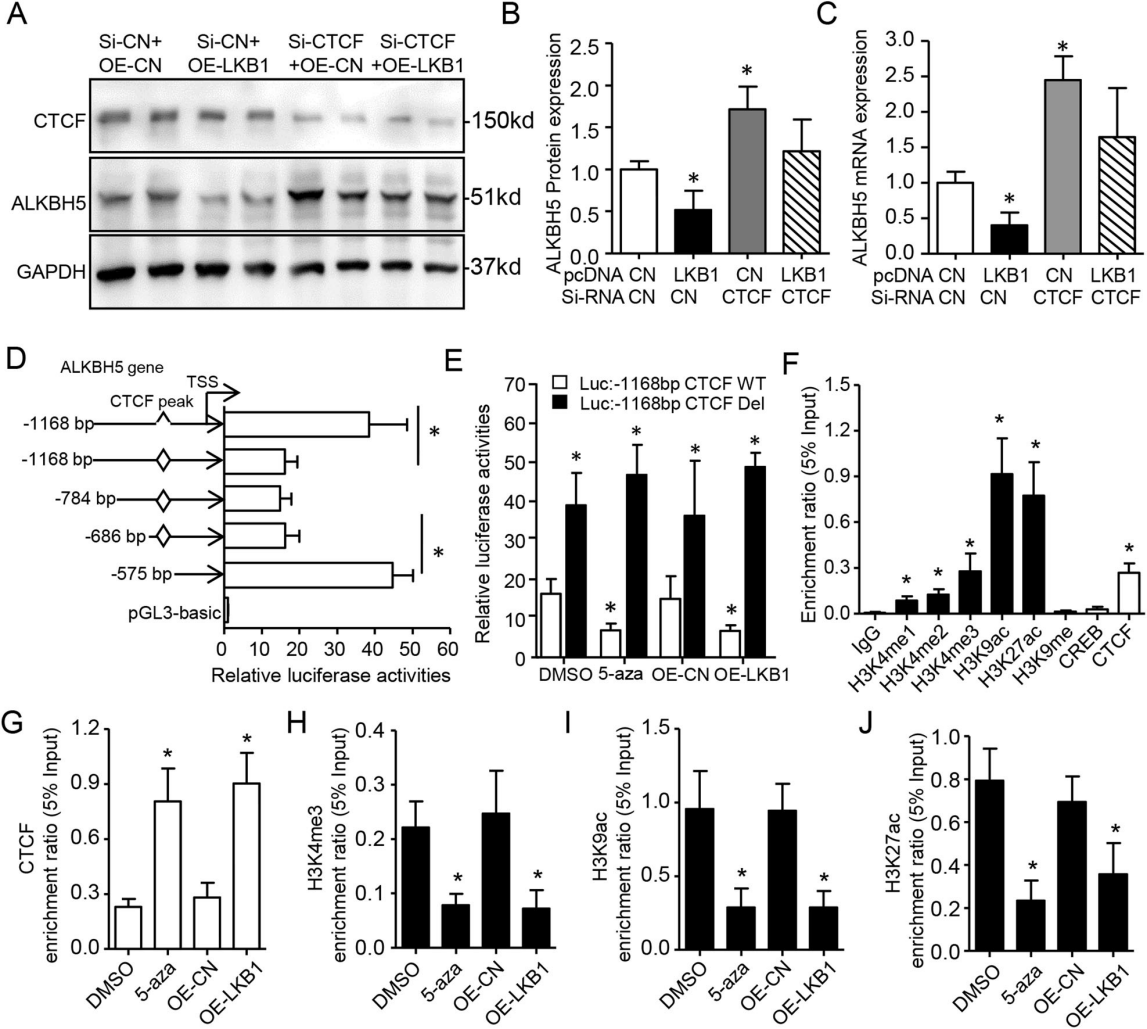
Suppressor-CTCF is required for *ALKBH5* downregulation by *LKB1*. **A**–**C** Representative images and quantification of CTCF and ALKBH5 protein expression by WB and qRT-PCR in A549 cells with LKB1 overexpression and/or CTCF knockdown for 48 h. Error bars, SD (*n* = 4 for WB; *n* = 5 for qRT-PCR), *P < 0.05 vs. Si-CN or OE-CN (one-way ANOVA with Bonferroni multiple comparison post hoc test). **D** Luciferase reporter assay of A549 cells transfected with pGL3-basic constructs containing serial LKBH5 promoters or deletion of CTCF peak fragment. *n* = 8/group, mean ± SD. * *P* < 0.05 by one-way ANOVA followed by Tukey’s test. **E** Reduction of *ALKBH5* Luc:-1168 bp wild-type (WT) construct activities by treatment of 5-aza or LKB1 overexpression in A549 cells, but not for *ALKBH5* Luc:-1168 bp deletion (Del). Data as mean ± SD (*n* = 5), *P < 0.05 (Student’s *t*-test). **F** ChIP-qPCR showing that the *ALKBH5*-CTCF peak region was occupied by the suppressor of CTCF and activators of histone modulators in A549 cells. **G**–**J** ChIP-qPCR analyses of 5-aza treated or LKB1 overexpressed A549 cells. *n* = 5/group, mean ± SD. *P < 0.05 vs. IgG in **F**, vs. A549-DMSO in **G**–**J** by one-way ANOVA followed by Tukey’s test.

To determine whether CTCF directly represses *ALKBH5*, we used western blot and RT-qPCR. We found that silencing CTCF increased *ALKBH5* protein and mRNA expression, and also rescued ALKBH5 repression by *LKB1* overexpression (Fig. 5A–C). We next generated serial deletions, including CTCF motif deletion constructs based on the human *ALKBH5* promoter, for the luciferase reporter assay. The CTCF-containing construct had lower *ALKBH5* promoter activity than constructs that lacked the motif (Fig. 5D). Notably, CTCF motif deletion increased *ALKBH5* promoter activity to levels similar to those in constructs without the CTCF motif. This evidence suggests that CTCF directly represses ALKBH5 transcriptional activity.

Next, we found that inhibition of DNA methylation by 5-aza treatment or by LKB1 overexpression reduced *ALKBH5* promoter activity on the CTCF motif-containing construct, but had no effect on CTCF motif-deficient construct (Fig. 5E). Further ChIP-qPCR analysis indicated that histone activators, such as H3K4me1, H3K4me2, H3K4me3, H3K9ac, and H3K27ac, occupy the ALKBH5 gene promoter (Fig. 5F), consistent with ChIP-seq results (Fig. S6). Interestingly, 5-aza treatment or LKB1 overexpression promoted CTCF enrichment on *ALKBH5*, and also partially prevented enrichment of H3K4me3, H3K9ac, and H3K27ac (Fig. 5H–J). Therefore, loss of LKB1 promoted DNA hypermethylation in the CTCF peak region, thus preventing CTCF binding and releasing repression of *ALKBH5* in *KRAS* mutant cells.

### ALKBH5 demethylation of m6A increased expression and stability of SOX2, SMAD7, and MYC in a YTHDF2-dependent pathway

**Fig. 6:**
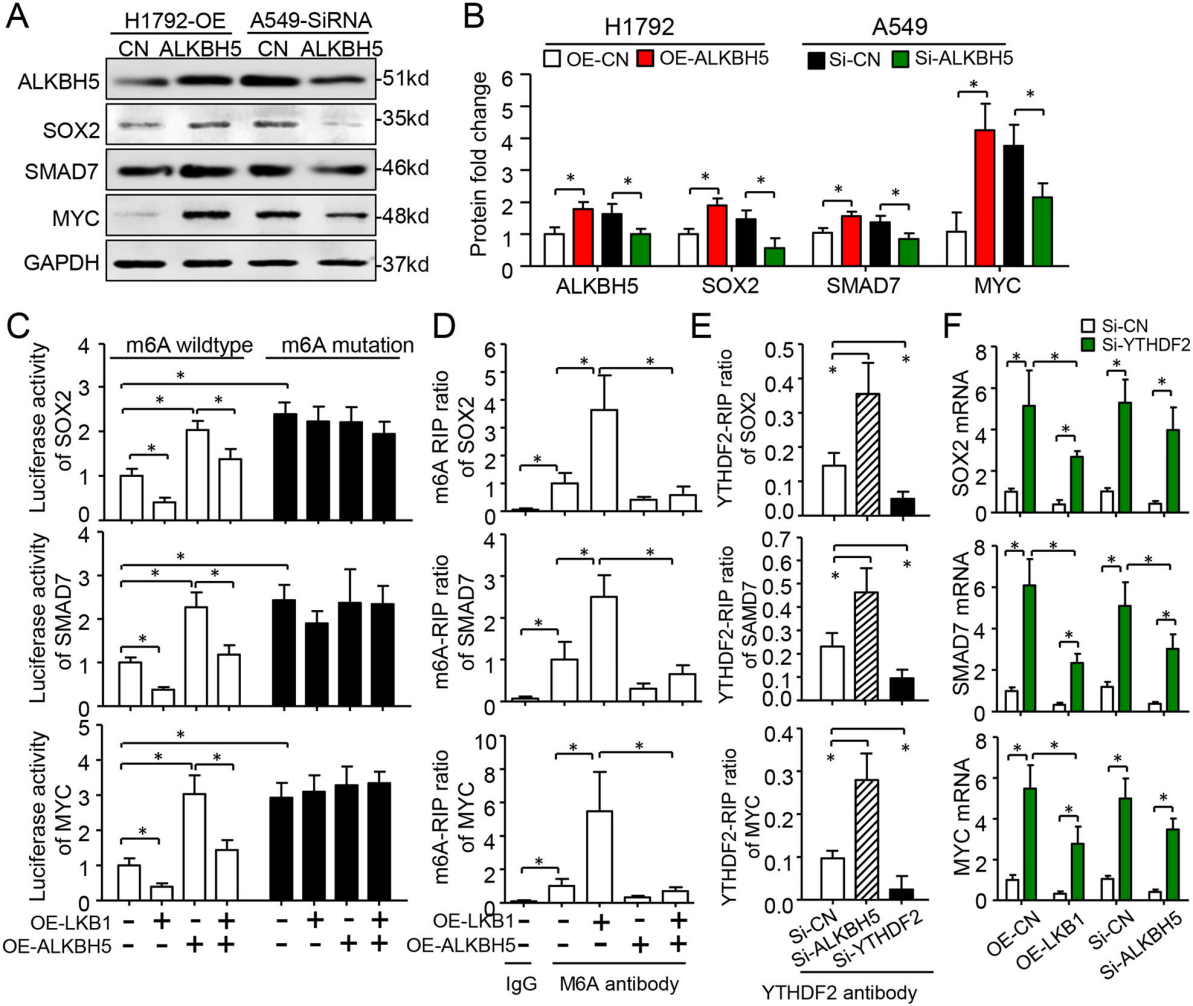
ALKBH5 erases m6A modification on *SOX2, SMAD7*, and *MYC* mRNA to increase their stability and expression. **A**, **B** Representative western blotting and quantification of SOX2, SMAD7, MYC, and ALKBH5 protein. Data as mean ± SD (*n* = 4). **C** Mutation of m6A site or ALKBH5 overexpression released the *SOX2, SMAD7*, and *MYC* gene posttranscriptional repression by LKB1 in A549 cells (*n* = 4). **D** m6A-RIP analysis demonstrated that *SOX2, SMAD7*, and *MYC* were subjected to ALKBH5-mediated m6A modifications (*n* = 5). **E** YTHDF2-RIP-qPCR shown that YTHDF2 could occupy m6A sites of *SOX2, SMAD7*, and *MYC*, which was mediated by ALKBH5 (*n* = 4). **F** qRT-PCR was performed to indicate that knockdown of YTHDF2 significantly upregulated *SOX2, SMAD7*, and *MYC* gene mRNA expression at basal level, LKB1 overexpressed or ALKBH5 silenced A549 cells (*n* = 6). Data as mean ± SD. *P < 0.05 by one-way ANOVA followed by Bonferroni multiple comparison post hoc test for **B**–**F**. Si-CN, Si-RNA-A. OE-CN, OE-c-Flag pcDNA3. OE overexpression.

To identify downstream targets of ALKBH5-mediated m6A modification in lung cancer, we overlapped 2605 genes using the canonical m6A motif-enriched gene stop codon region based on m6A-sequence in A549 cells (16) (Fig. S7A). Gene ontology analysis revealed that the 2605 genes were significantly enriched in gene transcription regulation, mRNA splicing, cell cycle, and mRNA stability (Fig. S7B). KEGG pathway analysis revealed that the overlapping genes were closely associated with Hippo and TGF-β pathways (Fig. S7C). Using qRT-PCR, we confirmed that 45.2% (14/31) of Hippo-Yap pathway genes were directly regulated by *ALKBH5* (Fig. S7D, E). Western blot assay further confirmed that *ALKBH5* regulated *SOX2*, *SMAD7*, and *MYC* proteins in A549 and H1792 cells (Fig. 6A, B).

To investigate m6A modifications on *SOX2, SMAD7*, and *MYC* mRNA, we used firefly luciferase assay (Fig. 6C). We found their activities were significantly less than those of their mutant reporters, indicating that m6A represses gene activity. Moreover, overexpression of *ALKBH5* increased luciferase activities of three m6A WT gene reporters, and also rescued the repressive activity mediated by *LKB1*-transfection. However, we observed no such effects for m6A mutant gene-fused reporters. Our m6A-RIP-qPCR assay showed significantly higher accumulation of m6A at mRNA fragments of all three genes than for the IgG control. However, this m6A enrichment was reduced by *ALKBH5* overexpression. Also, *ALKBH5* upregulation inhibited the increased m6A occupancy caused by *LKB1* overexpression (Fig. 6D). Given that *ALKBH5* overexpression or knockdown did not change the pre-mRNAs of *SMAD7* and *MYC* (Fig. S8A–D), we predicted m6A reader protein YTHDF2 regulated their mRNA stability. Interestingly, actinomycin-D induced degradation of *SMAD7*, *SOX2*, and *MYC* mRNAs was partially prevented by silencing YTHDF2 in both A549 and H1792 cells. (Fig. S8E–J). RIP-qPCR assay showed that YTHDF2 also occupied *SMAD7, SOX2*, and *MYC* m6A regions, and this enrichment increased in the absence of ALKBH5 (Fig. 6E). Furthermore, YTHDF2 silencing rescued their gene expression upon LKB1 overexpression or ALKBH5 knockdown (Fig. 6F). Thus, ALKBH5-m6A-YTHDF2 signaling prevented *SOX2, SMAD7*, and *MYC* mRNA decay.

### Confirmation of an axis of “LKB1-DNA methylation-ALKBH5-m6A” in clinical KRAS-mutated lung cancer patients

**Fig. 7:**
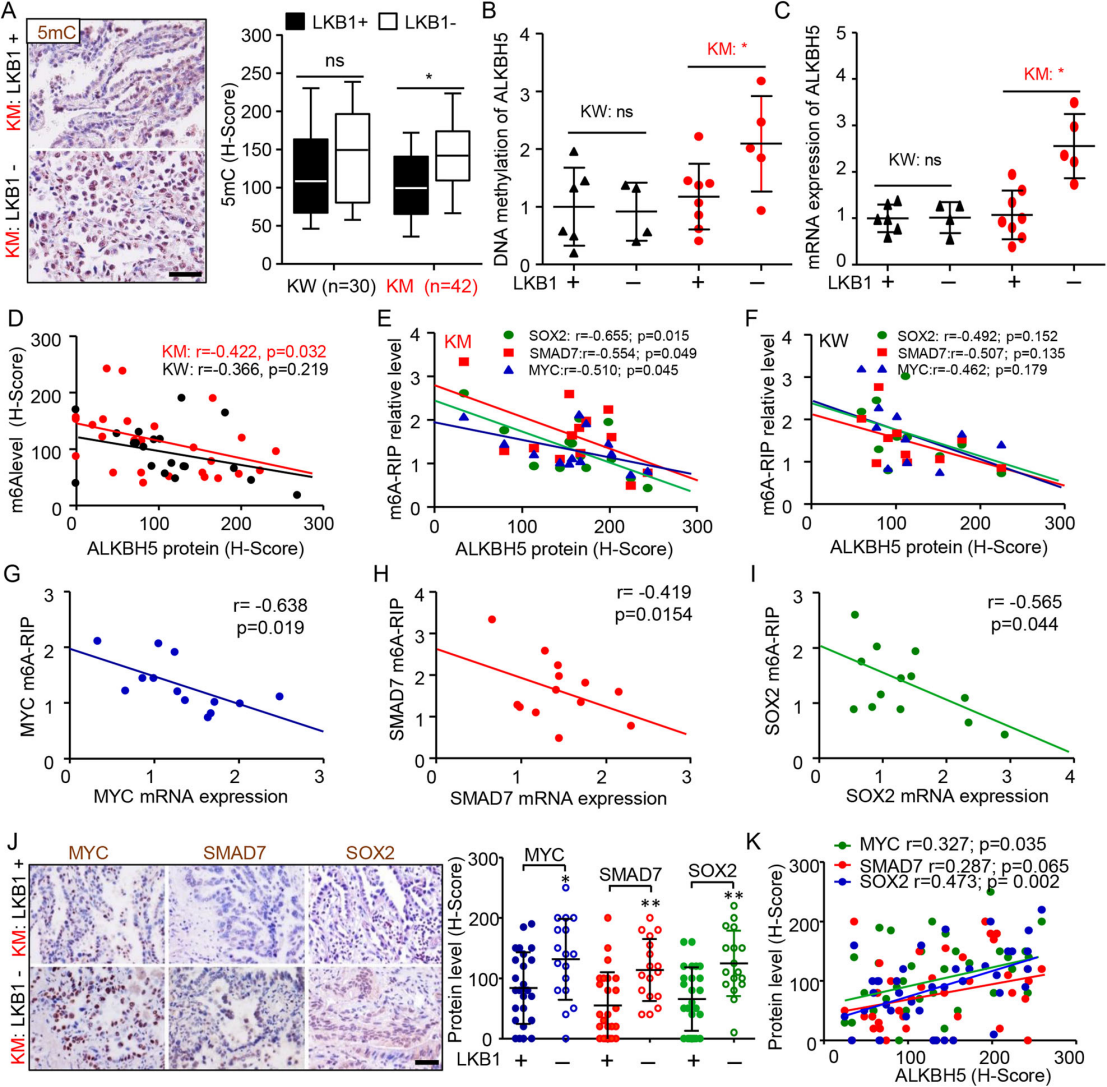
Loss of LKB1 clinically associates with the increase of ALKBH5 and decrease of m6A modification on *SOX2*, *SMAD7*, and *MYC* genes. **A** Representative immunostaining images and quantification of 5mC DNA in lung cancer patients with KW and KM. Boxes and whiskers represent the 10th to 90th percentiles, respectively; the median is the central line in each box. Bar = 50 µm. **B**, **C** High levels of *ALKBH5* DNA methylation and mRNA expression in cases of LKB1 loss compared with that of LKB1 positive expression in KM patients. **D**–**F** The correlation of ALKBH5 protein with the global m6A modification (**D**), m6A levels of *SOX2, SMAD7*, and *MYC* genes (**E**, **F**) in KM and KW groups. **G**–**I** Negative correlations of the m6A enrichment with mRNA expressions of *SOX2*, *SMAD7*, and *MYC* in KM group. **J** Representative immunostaining images and quantification of *SOX2*, *SMAD7*, and *MYC* in KM group with LKB1 loss or not. **K** The positive correlations of ALKBH5 with *SOX2, SMAD7*, and *MYC* protein expression in KM group. Data as mean ± SD. *KRAS* mutation (KM). *KRAS* wild-type (KW). *P < 0.05 by one-way ANOVA followed by Bonferroni multiple comparison post hoc test for **A**, **B**, and **C**. Student’s *t-*test for **J**.

Next, we validated the underlying regulation of m6A by LKB1 using a panel of clinically relevant lung adenocarcinoma specimens. IHC staining showed that LKB1 loss increased the global 5-mC DNA methylation in lung cancer tissues with *KRAS* mutations, but not in tissues with WT *KRAS* (Fig. 7A). Similar pattern was found for both DNA methylation and mRNA expression of *ALKBH5* (Fig. 7B, C). ALKBH5 protein expression negatively correlated with global m6A level, as well as the m6A levels for *SOX2, SMAD7*, and *MYC* genes only in *KRAS* mutant tissues (Fig. 7D–F). Furthermore, m6A levels of these three genes were negatively correlated with their mRNA expression (Fig. 7G–I). Notably, the protein levels of *SOX2, SMAD7*, and *MYC* were significantly increased by loss of LKB1 and correlated with ALKBH5 expression in *KRAS* mutant tissues (Fig. 7J, K). Thus, our clinical data further confirmed that LKB1 loss increased the DNA methylation and expression level of *ALKBH5*, which resulting in the demethylation of m6A modification on *SOX2, SMAD7*, and *MYC*, and maintaining their expression for KRAS mutant lung cancer.

## Discussion

**Fig. 8:**
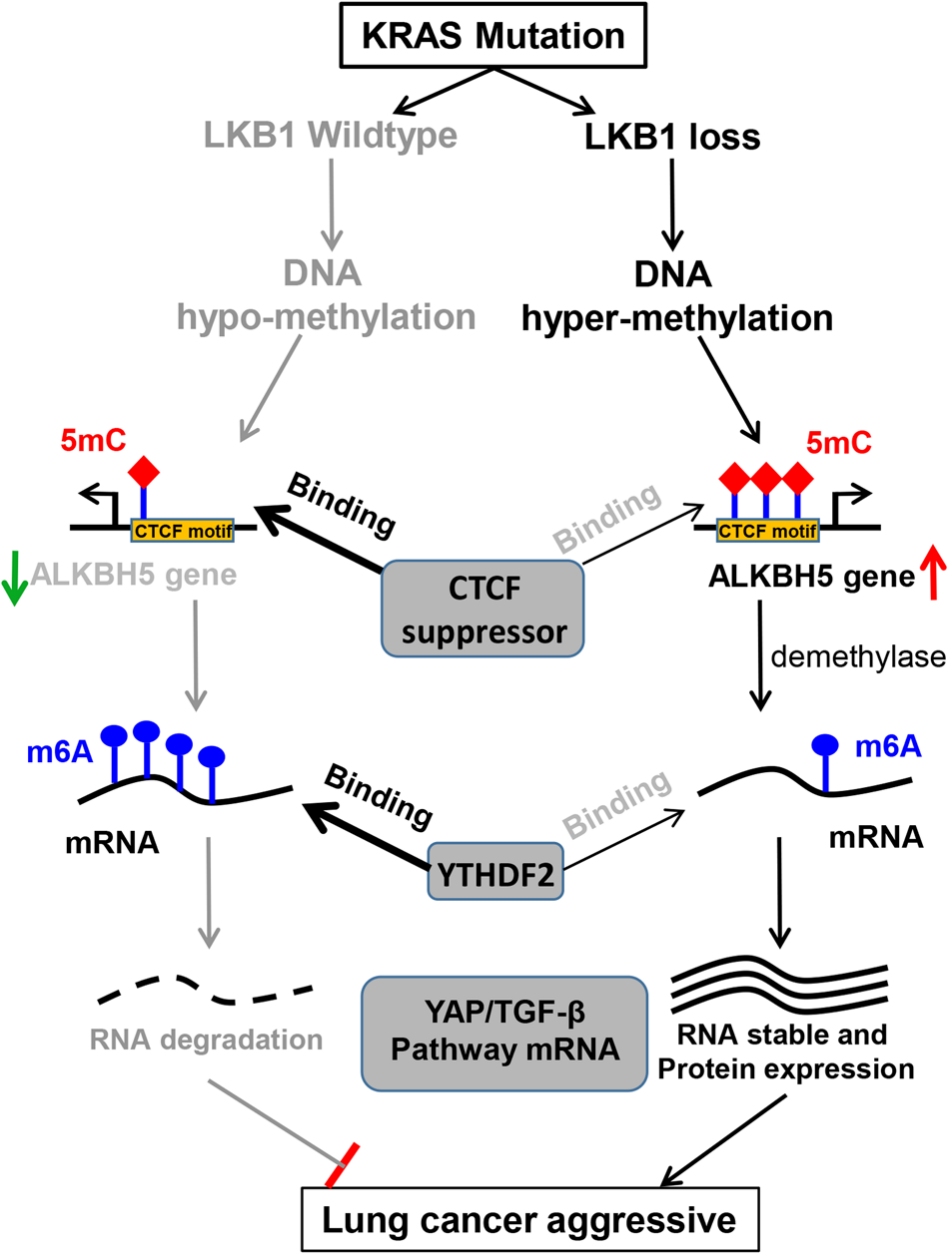
Proposed model for LKB1-mediated m6A modification in KRAS-mutated lung cancer progression. Loss of LKB1-induced DNA hypermethylation, which prevents CTCF binding on the *ALKBH5* gene promoter, maintains ALKBH5 expression, and further represses global RNA methylation. Oncogenic *SMAD7, SOX2*, and *MYC* are crucial targets of m6A meditated by LKB1 deficiency, and are involved in KRAS mutation lung cancer progression.

Our findings describe m6A RNA modification by 5mC-DNA mechanism in KL tumors that supports tumorigenic growth and progression (Fig. 8). Here, we report that reduced m6A RNA modification increased stability and expression of critical oncogenes and contributed to aggressive cancer phenotypes. Loss of LKB1 specifically enhanced ALKBH5 expression and reduced m6A levels in KRAS-mutated cells. LKB1 inactivation could increase the 5-mC DNA methylation of the *ALKBH5* promoter, which prevents CTCF binding and releases *ALKBH5* suppression. Subsequently, reduced “m6A-YTHDF2” signaling promoted the expression and stability of critical tumor oncogenes, such as *SOX2, SMAD7*, and *MYC*. Our study indicates that LKB1 loss reduces m6A modification by upregulating ALKBH5, which contributes to aggressive tumor progression and poorer outcomes for KL lung cancer.

Aberrant global m6A abundance has been increasingly reported in human cancers, and associates with cancer progression and clinical outcome^25^. Interestingly, m6A hypomethylation was reported in glioblastoma, bladder cancer, endometrial cancer^26^, melanoma, or breast carcinoma, whereas, m6A hypermethylation was found in gastric cancer and hepatocellular carcinoma^27^. Writing and erasing proteins regulate m6A levels, which enable the binding of m6A reader proteins and initiate a series of biological functions. For example, ALKBH5 demethylates m6A mRNAs, which modulates mRNA splicing, export, and stability. ALKBH5 deficiency leads to aberrant spermatogenesis and apoptosis in mouse testes, likely through regulating genes associated with the p53 network^22^. ALKBH5 often exerts an oncogenic role in GBM, pancreas, cervical, and breast cancer, but acts a tumor-suppressor in leukemia^25^. This two-sided role of ALKBH5, might relate with the complex and diverse function of m6A modification, which not only promotes the translation of related mRNAs but also reduces the mRNAs stability by binding with different reader proteins^13–16^. Thus, It would be important to determine how critical role of m6A and its regulator in each type of cancer.

Limited literature is available to explain how m6A regulators could be modulated in cancers, although many studies have described roles for m6A regulators in cell fate and carcinogenesis^20,28,29^. This study identifies ALKBH5 as a functional target gene of 5-mC DNA methylation, which controls m6A RNA modification. Notably, ALKBH5 is one of the top five 5-mC DNA methylation biomarkers that helped distinguish patients with metastatic-lethal prostate cancer^30^, suggesting that DNA methylation of *ALKBH5* may be a prognostic indicator. We also identified CTCF as an effective transcriptional suppressor that was highly sensitive to global 5-mC DNA methylation and required for *ALKBH5* repression. Similarly, we previously showed that DNA hypomethylation facilitated CTCF binding to and suppression of *hTERT* expression in human endothelium^31^. Recent studies show 5-mC DNA hypermethylation facilitated tumorigenesis in *KRAS/LKB1* co-mutated cancer^11^. LKB1 inactivation causes DNA hypermethylation and histone methylation, which facilitates immune escape in KL-mutated lung cancer and represses anti-oncogenic *STING*^32^. Consistent with those previous findings, our work indicates that oncogenic ALKBH5 is another target of LKB1 by a similar mechanism. Thus, loss of LKB1 reduces m6A modification also might via the linking of 5mC-DNA and histone modification in KL cancer.

Consistent with previous studies, our observations indicate that *ALKBH5* is a lung cancer oncogene because it promoted cell proliferation and migration^18,21^. Increased ALKBH5 expression predicted poor survival in our analysis. Moreover, we identified three critical oncogenes, *SOX2, SMAD7*, and *MYC*, as targets of ALKBH5-mediated m6A modification. Mechanistically, LKB1 loss or ALKBH5 overexpression increased *SAMD7*, *SOX2*, and *MYC* stability via a YTHDF2-dependent mechanism. This result is supported by previously reported PAR-CLIP-Seq data in a Hela cell line^13^. YTHDF2-induced decay of m6A modified genes may be a common pathway across cell types. We also found that YTHDF1 occupied and promoted *SAMD7* or *MYC* mRNA translation in an m6A-dependent manner^33^. Thus, it would be interesting to identify ALKBH5 targets by comparing the proteomic changes based on the differential level of ALKBH5.

Notably, LKB1 regulation of *ALKBH5* via 5mC-DNA was not limited to *KRAS* mutant lung cancer, but extended to pancreatic and colorectal cancer, which are the top three causes of cancer death in the United States^34^. Aggressive lung tumorigenesis, tumor progression, and poor prognosis were observed in mice with *Kras* mutation combined with *Lkb1* inactivation^8^. This tumor type is largely resistant to both standard-of-care treatments like docetaxel and combination treatment with a MEK inhibitor^5,35^. Co-mutations were also associated with an inert tumor immune microenvironment and poor clinical response to immune checkpoint blockade^36–38^. Thus, understanding molecular mechanisms may improve therapeutic strategies for cancer with *KRAS* and *LKB1* co-mutations. Screening chemicals that may regulate m6A formation or removal is an effective approach for developing tumor therapeutics. For example, R-2HG, an ALKBH5 and FOT inhibitor, inhibits leukemia cell growth and induces apoptosis in mice^39^. m6A-YTHDF2 inactivity contributes to melanoma progression by enhancing the expression and stability of key immune checkpoint factors, including PD-1, CXCR4, and SOX10^40^. This implies that YTHDF2 modulation could be combined with an anti-PD-1/PD-L1 blockade to improve anticancer immunotherapy. In support of this idea, we identified the ALKBH5-m6A-YHTDF2 axis as a targetable molecular pathway to treat this aggressive cancer.

In conclusion, our results uncover the epigenetic reprogramming of DNA methylation and m6A RNA methylation during *KRAS* mutation/LKB1 loss induced lung carcinogenesis. We also reveal that histone modification was necessary for ALKBH5 upregulation, which in turn controlled m6A RNA modification. Our findings and other recent reports^10,14,15,17^ highlight the important linking of the global DNA methylation and m6A RNA modification in cancer. This epigenetic reprogramming indicates new therapeutic approaches to tumorigenesis, especially for the lung cancer with dual *KRAS* mutation/LKB1 loss.

## Materials and methods

### Human lung cancer specimens and cell lines

We randomly obtained fresh and paraffin-embedded lung adenocarcinoma specimens from 72 patients who underwent tumor resection surgery between January 2016 and September 2019 at the Cancer Hospital of Harbin University Medical College, China. Two pathologists performed blinded histological confirmation by hematoxylin and eosin (H&E) staining. KRAS mutations were detected by Cobas KRAS Mutation Test, which could detect 19 mutation sites based on a real-time PCR method. Exclusion criteria were patients who had received preoperative chemotherapy, radiation therapy, or had multiple metachronous or metastatic lesions. Clinical characteristics of patients were retrospectively analyzed and summarized in Table S1. We collected all clinical samples with informed consent according to Health Insurance Portability and Accountability Act (HIPAA)-approved protocols. This study was approved by the ethical review board of Cancer Hospital of Harbin University Medical College on October 22, 2018. All of the patients were given and accepted informed consent form prior to their enrollment.

MRC-9, H1299, H1650, H1703, H1795, H1792, A549, DLD-1, SW480, Panc-1, and MIA PaCa-2 cell lines were purchased from ATCC, USA. Cells were cultured in medium according to the manufacturer’s instructions and grown in a humidified incubator at 5% CO_2_. All cell lines were authenticated and confirmed negative for mycoplasma contamination by providers.

### RNAi and protein overexpression transfection

To knockdown endogenous gene expression, we purchased si-RNAs targeting human *LKB1* (sc-35816), *ALKBH5* (sc-93856), *YTHDF2* (sc-78661), and *CTCF* (sc-35124) from Santa Cruz and transiently transfected these si-RNAs into lung cancer cells for 48 h using Lipofectamine RNAiMAX (Invitrogen). We ordered LKB1 (#8590) and ALKBH5 (#38073) overexpressing vectors from Addgene and transfected them into human cancer cells using Lipofectamine 2000 (Invitrogen) for 24 h. Si-RNA-A (sc-37007, Santa Cruz) and c-Flag pcDNA3 (Plasmid #20011, Addgene) were served as transfect controls for genes knockdown and overexpression, respectively.

### Quantitative reverse transcription polymerase chain reaction (qRT-PCR)

We extracted total cellular and tissue RNA using TRIzol Reagent (Thermo Fisher Scientific, USA) and used 1 µg total RNA for reverse transcription using the iScript^™^ cDNA Synthesis Kit (Bio-Rad). qRT-PCR was performed with 2× SYBR Green qPCR Master Mix (Bimake, USA) with a *CFX96* Touch^™^ Real-Time PCR detection system (Bio-Rad Inc. USA). We calculated the relative gene expression using the comparative CT method and β-actin RNA sequences as a control. Primer sequences were listed in Table S2.

### Western blot analysis

We lysed total protein from treated cells using RIPA buffer (Cell Signaling Technology) supplemented with Protease Inhibitor Cocktail (Thermo Fisher; 78430). Western blotting was performed as previously described^41^, and the primary antibodies included GAPDH (Santa Cruz, sc-137179), LKB1 (Santa Cruz, sc-32245), ALKBH5 (Proteintech, 16837-1-AP), CTCF (Santa Cruz, sc-271474), METTL3 (Abcam, ab195352), METTL14 (Abcam, ab220030), FTO (Abcam, ab126605), WTAP (Proteintech, 60188-1-Ig), SOX2 (Cell signaling, #3579), SMAD7 (Santa Cruz, sc-11392), and MYC (Cell signaling, #13987). We performed densitometric analyses of band intensity using ImageQuant TL 8.2 image analysis software (GE Healthcare Life Sciences, USA) and GAPDH was as an internal control.

### Immunohistochemistry (IHC) staining

IHC analysis of paraffin-embedded lung cancer tissues containing primary tumors and matched normal lung tissues as previously described^42,43^. In brief, we de-paraffinized and rehydrated human tissue sections to retrieve antigens, and then incubating in 1% hydrogen peroxide. After blocking, we applied primary antibodies to the slides at 1:500 (anti-LKB1, METTL3, METTL14, WTAP, and FTO antibodies), 1:200 (anti-ALKBH5 antibody), and 1:1000 (anti-m6A and 5-mC antibody) dilutions and incubated at 4 °C overnight. We stained slides with EnVision+ Dual Link System-HRP (Dako) for 1 h at room temperature. Images of stained cells in four random fields were captured by using an optical microscope (Olympus, Japan). Relative protein expression was evaluated by a Histoscore (H-score) system. The results were evaluated by two independent pathologists.

### Immunofluorescence staining

We performed immunofluorescence staining on cultured A549 cells transfected with siRNA or pcDNA. After treatment, we fixed cells with 4% paraformaldehyde (PFA), permeabilized with 0.3% Triton/phosphate-buffered saline (PBS), blocked in 1% bovine serum albumin (BSA), and then incubated with the indicated primary antibody for LKB1 (Santa Cruz, sc-32245), ALKBH5 (Proteintech, 16837-1-AP), or m6A (Synaptic Systems, 202111) overnight at 4 °C. The next day, cells were washed and incubated with secondary antibody conjugated to Alexa Fluor 555 (donkey anti-rabbit, Invitrogen) for 1 h at room temperature. We used DAPI (Sigma) staining to label nuclei. Fluorescence was observed under a Leica SP8 confocal laser scanning microscope.

### Luciferase reporter assays

For the *ALKBH5* promoter reporter assay, we generated serial ALKBH5 promoter reporters by PCR amplification and inserted into pGl3-basic plasmid, as we previously reported^41^. We deleted the *CTCF* region using the Q5 Site-Directed Mutagenesis Kit (NEB) per the manufacturer’s protocol. Firefly luciferase activity was used to evaluate the effect of m6A modification on *SOX2, SMAD7*, and *MYC* activation. We used the pmirGLO Dual-Luciferase miRNA target expression vector from Promega to construct the reporter plasmid, which contained both a firefly luciferase and a Renilla luciferase. We constructed mutant three reporter plasmids by replacing the adenosine bases within the m6A consensus sequences with cytosine. All constructs were confirmed by Sanger sequencing. Nucleotide sequences of primers were in Table S2. A549 cells grown in 96-well plates were transfected with reporter vectors and SV-40-Renilla-Luc in the presence of Lipofectamine 2000 Reagent (Invitrogen). Luminescence was measured with the Dual-Luciferase Reporter Assay System (Promega). We performed experiments for each vector as biological triplicates with six technical repeats.

### Global 5-mC and m6A measurement

We measured total cellular and tissue 5-mC in DNA and m6A in mRNA levels by the MethylFlash Methylated DNA 5-mC Quantification Kit (Colorimetric) and EpiQuik m6A RNA Methylation Quantification ELISA kit (Colorimetric) (Epigentek Group Inc.), respectively. Total genomic DNA and RNA were isolated using the PureLink Genomic DNA Mini Kit and TRIzol Reagent (Thermo Fisher Scientific). We used 200 ng of DNA or RNA for additional global 5-mC and m6A measurement according to the manufacturer’s instructions. Measurements were performed in triplicate.

### Bisulfite genome sequencing (BGS)

For DNA methylation analysis, we used BGS method, as previously described^31,44^. Briefly, 500 ng of genomic DNA was bisulfite converted using the BisulFlash DNA Modification Kit (Epigentek) following the manufacturer’s instructions. We amplified the fragment containing CTCF peak region of ALKBH5 promoter using primers listed in Table S2. PCR products were purified and cloned into a pCR4 TOPO vector using the TOPO TA Cloning Kit (Thermo Fisher Scientific, Rockford, IL, USA). We isolated and sequenced plasmid DNA from ten randomly selected clones (Genewiz, Piscataway, NJ, USA).

### Methylated DNA immunoprecipitation (MeDIP) analysis

To confirm BGS results, we performed MeDIP using the Methylamp Methylated DNA Capture Kit (EpiGentek) according to the manufacturer’s instructions. Briefly, we extracted cellular and tissue chromatin DNA and digested to ~150–700 bp using a micrococcal nuclease (CST). The fragmented DNA was immunoprecipitated with anti-5-mC (Abcam, ab10805) at room temperature for 2 h. After washing and purifying the DNA, we quantified the methylation status using qPCR. Primers were listed in Table S2.

### m6A-RNA Immunoprecipitation (m6A-RIP)

We extracted total RNA from treated A549 cells or tumor tissues, and incubated with DNase according to the TURBO DNA-free TM Kit (Thermo Fisher) protocol to avoid DNA contamination. Then, we chemically fragmented 1 µg/µl RNA and incubated with m6A antibody to immunoprecipitate according to the standard protocol for the EpiMark *N*6-Methyladenosine Enrichment Kit (NEB). Enrichment of m6A containing mRNA was then analyzed using qRT-PCR. Primers targeting m6A-enriched regions of *SOX2*, *SMAD7*, and *MYC* were listed in Table S2.

### Cell proliferation and migration assay

For cell colony formation assay, A549 or H1792 cells were transfected with siRNA or pcDNA for 24 h and seeded into 6-well plates (500/well). After 1 week, we fixed formed colonies and stained with 0.1% crystal violet in 20% methanol, and counted colonies consisting of at least 50 cells. For the migration assay, we resuspended 5 × 10^5^ cells in Opti-MEM Reduced Serum Media (Invitrogen) and seeded into the upper chamber of a transwell apparatus (8.0 μm, BD Biosciences), and added complete medium to the bottom chamber to provide chemoattractants for migration. To count migrated cells, we captured images of stained cells in five random fields by using an optical microscope (Olympus, Japan) and counted samples in triplicate.

### Bioinformatics assay from database

The differentially expressed genes (DEGs) of m6A modulators (*METTL3, METTL14, WTAP, FTO*, and *ALKBH5*) and readers (*YTHDF1, YTHDF2, YTHDF3, YTHDC1*, and *YTHDC2*) between KL and K-lung cancer cell lines or tissues from the databases of the Cancer Genome Atlas (TCGA, https://portal.gdc.cancer.gov/), Mouse Tumor Biology (MTB, http://tumor.informatics.jax.org/mtbwi/index.do), and Cancer Cell Line Encyclopedia (CCLE, https://portals.broadinstitute.org/ccle/home).

### m6A-seq data analysis

Based on the NCBI-GEO database (GEO: GSE76367), we determined the number of m6A-seq fragments mapped to each gene using HT-Seq. We overlapped the core m6A peaks based on functional m6A signal enriched around the stop codon (−5 to +5 kbp) of mRNAs and canonical m6A peaks^45,46^. Then, core m6A peaks were subjected to functional enrichment analysis by Ingenuity Pathway Analysis (IPA) (http://www.qiagen. com/ingenuity) and KEGG pathway (https://www.genome.jp/kegg/).

### Statistics

All experiments were repeated at least four times, unless otherwise stated in the figure legend. We performed statistical analyses using the SPSS v20.0 software (SPSS Inc., Chicago, IL), and used Student’s *t*-test (two-tailed) or one-way ANOVA analysis followed by Tukey’s, Sidak’s, or Bonferroni test to assess statistical significance between or among groups. We calculated the survival rate using the log-rank (Mantel–Cox) test. Normality was assumed and variance was compared between or among groups. All numerical data were presented as mean ± standard deviation (SD) and a *p* value of <0.05 was considered significant.

## Supplementary information

Supplementary table Legends

Supplementary Figure Legends

Supplement Tables

Supplementary figure 1

Supplementary figure 2

Supplementary figure 3

Supplementary figure 4

Supplementary figure 5

Supplementary figure 6

Supplementary figure 7

Supplementary figure 8

Supplementary figure 9

Supplementary figure 10

Supplementary figure 11

Supplementary figure 12

Supplementary figure 13

Supplementary figure 14
