# Supplementary material for: Suppression of m6A mRNA modification by DNA hypermethylated ALKBH5 aggravates the oncological behavior of KRAS mutation/LKB1 loss lung cancer
Source: Cell Death Dis. 2021 May 20;12(6):518. doi: 10.1038/s41419-021-03793-7 (PMC8137886; doi:10.1038/s41419-021-03793-7)
Supplement: Supplementary file 1 — Supplementary table Legends [file 41419_2021_3793_MOESM1_ESM.docx]

**Supplementary Table Legends**

**Table S1. Clinic-pathological variables of lung cancer patients.**

Note: KRAS ^Wt^ LKB1 ^Wt^, WT; KRAS ^Wt^ LKB1 ^Loss^, L; KRAS ^Mut^ LKB1 ^Wt^, K; KRAS ^Mut^ LKB1 ^Loss^, KL.

**Table S2. Complete list of primers used in this work. Sequences are from the GenBank.**

Note: * P1, Primer1. ^#^P2, Primer2.
