# Supplementary material for: Suppression of m6A mRNA modification by DNA hypermethylated ALKBH5 aggravates the oncological behavior of KRAS mutation/LKB1 loss lung cancer
Source: Cell Death Dis. 2021 May 20;12(6):518. doi: 10.1038/s41419-021-03793-7 (PMC8137886; doi:10.1038/s41419-021-03793-7)
Supplement: Supplementary file 2 — Supplementary Figure Legends [file 41419_2021_3793_MOESM2_ESM.pdf]

Supplementary Figure Legends

Figure S1. ALKBH5 is dispensable for the aggressive lung cancer with KRAS wildtype. (A) Quantification of LKB1 and m6A modulators in lung cancer patients with KRAS wildtype. (B) Spearman correlation between ALKBH5 expression and m6A level. (C) The relationship between of ALKBH5 and the clinical characters of KRAS wildtype lung cancer patients. r value are calculated by Spearman correlation. *P < 0.05. (D) The m6A level and ALKBH5 expression in KRAS wildtype lung cancer patients with TTF-1 positive or negative. Boxes and whiskers represent the 10th to 90th percentiles, respectively; the median is the central line in each box. ***P < 0.001 by Student's t test. ns, not significant by Student's t test. WT, LKB1 ^{WT} KRAS ^{WT}; L, LKB1 ^{Loss} KRAS ^{WT}.

Figure S2. LKB1 loss associates with ALKBH5 up-regulation in KRAS^{mut} lung cancer. The differentially expressed genes (DEGs) of LKB1, m6A modulators and readers in KL related with K of human lung cancer tissues from the Cancer Genome Atlas (TCGA) database (A), Cancer Cell Line Encyclopedia (CCLE) database (B), as well as in KL related with K of mice lung cancer tissues from Mouse Tumor Biology (MTB) database (C). (D) The correlations of mRNA expression between LKB1 and m6A modulators or readers from TCGA, CCLE and MTB databases. Data as mean±SD. *P < 0.05. KRAS ^{Mut} LKB1 ^{WT} (K), KRAS ^{Mut} LKB1 ^{Loss} (KL).

Figure S3. The regulation of m6A modulators by LKB1 in KRAS mutant or wildtype lung cancer cells. (A) Western blot assay for LKB1 protein expression profiles. GAPDH was the internal control. (B-E) For KRAS mutant cells, ALKBH5 was upregulated by Si-LKB1 transfection in H1792 (B), and down-regulated by p-cDNA-LKB1 transfection in A549 cells for 48 hrs (C). No changes of DNMT1 and ALKBH5 in KRAS wildtype cells:

H1299 with LKB1 knockdown (D) and 1703 with LKB1 overexpression for 48 hrs (E). DNMT1 mRNA expression was used as positive control. Data as mean±SD (n=5). *P < 0.05 by Student's t test. CN, Control; OE, Over-expression.

Figure S4. LKB1 negatively regulated ALKBH5 in KRAS mutant pancreatic cancer and colorectal cancer. Pearson correlation analysis showing LKB1 negatively related with ALKBH5 in KRAS mutant lung cancer (A), pancreatic cancer (B) and colorectal cancer (C) cell lines from the CCLE database. qRT-PCR analysis shows that ALKBH5 mRNA was negatively regulated by transfection of Si-LKB1 or p-cDNA-LKB1 in the KRAS mutant pancreatic cell line: MIA PaCa-2 cells (D) and colorectal cancer line: SW480 (E). There was no altering of ALKBH5 by LKB1 knockdown or overexpression by KRAS wildtypes cell line PANC-1 and DLD1 cell lines (F,G). KW, KRAS wildtype; KM, KRAS mutant. Data as mean±SD (n=4). *P < 0.05, ** P < 0.01, *** P < 0.001 vs. OE-CN or Si-CN by Student's t test. CN, Control; OE, Over-expression.

Figure S5. ALKBH5 DNA methylation negatively relates with its mRNA expression in KRAS mutant cancers. Pearson correlation analysis showing ALKBH5 mRNA expression was negatively related with its DNA methylation in KRAS mutant (KM), but not wildtype (KW) of lung cancer (LC, A,B) and colorectal cancer (CRC, C,D) cell lines. Data was collected from the CCLE database.

Figure S6. The enrichment of transcriptional activators and suppressors on human ALKBH5 gene promoter by ChIP-sequence data in A549 cells from ENCODE database.

Figure S7. Identification of m6A target genes via ALKBH5. (A) Venn diagram indicating the 2605 core-m6A specific target genes. (B, C) Enriched function terms of core-m6A target genes by Gene Ontology and KEGG assay. (C, D) The changes of Hippo-Yap pathway genes expression at mRNA level by qRT-PCR assay in H1792 cells with ALKBH5 over-expression (D) and in A549 cells with ALKBH5 knockout (E). Data as mean±SD (n=4/group). *P< 0.05 vs. OE-CN in C; vs. Si-RNA-A by Student t-test.

Figure S8. YTHDF2 regulates the stability of SOX2, SMAD7 and MYC mRNAs. (A, B) Diagram of human SMAD7 (A) and MYC (B) pre-mRNA PCR primer binding sites. Only the intronic primer pair can bind to this species. (C and D) Comparison of SMAD7 and MYC pre-mRNA expression by ALKBH5 overexpression in H1792 cell (C) and ALKBH5 knockdown in A549 cell (D). (E-J) The stability of SOX2, SMAD7 and MYC mRNA by treated with actinomycin D in H1792 (E-G) and A549 (H-J) cells after transfected with Si-CN and Si-YTHDF2 into for 24 hrs. Relative mRNA levels were quantified by qRT-PCR. The data were presented as the mean±SD (n=4). *P< 0.05 vs. Si-CN by two-way ANOVA followed by Bonferroni multiple comparison post hoc test. ns, not significant using the Student's t test.

Figure S9: Whole blots of western blotting for Figure 2G.

Figure S10: Whole blots of western blotting for Figure 3A.

Figure S11: Whole blots of western blotting for Figure 4B.

Figure S12: Whole blots of western blotting for Figure 5A.

Figure S13: Whole blots of western blotting for Figure 6A.

Figure S14: Whole blots of western blotting for Figure S2A.
