# Supplementary material for: Suppression of m6A mRNA modification by DNA hypermethylated ALKBH5 aggravates the oncological behavior of KRAS mutation/LKB1 loss lung cancer
Source: Cell Death Dis. 2021 May 20;12(6):518. doi: 10.1038/s41419-021-03793-7 (PMC8137886; doi:10.1038/s41419-021-03793-7)
Supplement: Supplementary file 3 — Supplement Tables [file 41419_2021_3793_MOESM3_ESM.pdf]

Table S1.

Characters	WT	L	K	KL
Number	16	14	25	17
Age (Mean ± SD)	63.25±9.07	60.79±11.18	58.76±10.65	61.18±8.66
Gender (Male %)	43.75	42.85	44.00	41.18
Smoking (%)	31.25	50.00	48.00	41.06
Tumor size (mm^3)	12.12±20.00	3.23±3.23	13.79±27.89	38.54±55.36
Lymph node metastasis (%)	25.00	21.43	20.00	35.29
TTF-1 Positive (%)	62.50	50.00	68.00	35.29

Table S2.

Assay Name	Reverse primer sequence (5'–3')	Products
**RT–PCR for mRNA**		
LKB1	Forward: TCTACAACATCACCACGGGTC	146bp
	Reverse: TTCGTACTCAAGCATCCCTTTC	
Dnmt1	Forward: CCTAGCCCCAGGATTACAAGG	118bp
	Reverse: ACTCATCCGATTTGGCTCTTTC	
METTL14	Forward: AGTGCCGACAGCATTGGTG	101bp
	Reverse: GGAGCAGAGGTATCATAGGAAGC	
METTL3	Forward: TTGTCTCCAACCTTCCGTAGT	145bp
	Reverse: CCAGATCAGAGAGGTGGTGTAG	
FTO	Forward: AACACCAGGCTCTTTACGGTC	236bp
	Reverse: TGTCCGTTGTAGGATGAACCC	
ALKBH5	Forward: ATGCACCCCGGTTGGAAAC	250bp
	Reverse: GACTTGCGCCAGTAGTTCTCA	
WTAP	Forward: TTGTAATGCGACTAGCAACCAA	121bp
	Reverse: GCTGGGTCTACCATTGTTGATCT	
AXIN1	Forward: GGTTTCCCCTTGGACCTCG	157bp
	Reverse: CCGTCGAAGTCTCACCTTTAATG	
CCND1	Forward: GCTGCGAAGTGGAAACCATC	135bp
	Reverse: CCTCCTTCTGCACACATTTGAA	
ID2	Forward: AGTCCCGTGAGGTCCGTTAG	112bp
	Reverse: AGTCGTTCATGTTGTATAGCAGG	
SMAD3	Forward: CCATCTCCTACTACGAGCTGAA	149bp
	Reverse: CACTGCTGCATTCCTGTTGAC	
SMAD7	Forward: TTCCTCCGCTGAAACAGGG	116bp
	Reverse: CCTCCCAGTATGCCACCAC	
SOX2	Forward: GCCGAGTGGAAACTTTTGTCG	155bp
	Reverse: GGCAGCGTGTACTTATCCTTCT	
MYC	Forward: GGATTCCCGCCTCAGAATAAC	99bp
	Reverse: GTGGGTGTGGGTTGTTCAGG	
β-ACTIN	Forward: TTAGTTGCGTTACACCCTTTC	156bp
	Reverse: GCTGTCACCTTCACCGTTC	
**RT–PCR for Pre-mRNA**		
SMAD7-P1*	Forward: AAGGCTGGAAGGTCTCCGTA	260bp
	Reverse: GGGGCCCCACTAATCTACAG	
SMAD7-P2 [#]	Forward: GGGGTAGCCAGAGCATTGTT	295bp
	Reverse: GGATACACCACAACAGGGCA	
MYC-P1	Forward: TTTGCTTTGAACTCGCTGCC	141bp
	Reverse: GCTCTCGTTCCTCCCCAAC	
MYC-P2	Forward: GGCTGGTGGAGTGGTAGAGT	299bp
	Reverse: GCTTCCCTACATCCCAAACCT	
**LKB1 luciferase report assay**		
	Forward: GGGGTACCCCTGACTCGTGAAACGTCCGCT	1967bp
LKB1 -1168~+789bp	Reverse: CCGGGTGGGTTACCTGAGCACCTAGCTAGCTAG	
	Forward: GGGGTACCCCAAAGAAGTGGAAGCGCCAAAG	1583bp
LKB1 -784~+789bp	Reverse: CCGGGTGGGTTACCTGAGCAC CTAGCTAGCTAG	
	Forward: GGGGTACCCCCGGACGATGCCGTGACGCGG	1487bp
LKB1 -686~+789bp	Reverse: CCGGGTGGGTTACCTGAGCAC CTAGCTAGCTAG	
	Forward: GGGGTACCCC GAGAGCGTTCCCTTGAAGCC	1374bp
LKB1 -575~+789bp	Reverse: CCGGGTGGGTTACCTGAGCAC CTAGCTAGCTAG	
LKB1 -1168~+789bp	Forward: GTTGGCAATATGAGCGCGAGAGCGTTCCCTTG	1912bp
with CTCF peak Del	Reverse: CAAGGGAACGCTCTCGCGCTCATATTGCCAAC	
SMAD7 WT	Forward: CCGCTCGAG GGCTGCTGCATAAACTCGTG	485bp

	Reverse: GGAGTCACCAATGCCTGTCC GCTAGCTAG	
SMAD7 Mut	Forward: GAGAGGGCTCCTGGACACAGTAGAGCCTC<br>Reverse: GAGGCTCTACTGTGTCCAGGAGCCCTCTC	485bp
SOX2 WT	Forward: CCGCTCGAGGCAATAGCATGGCGAGCG<br>Reverse: GACTTGACCACCGAACCCATGCTAGCTAG	342bp
SOX2 Mut	Forward: GTGAACCAGCGCATGGTCAGTTACGCGCACATG<br>Reverse: CATGTGCGCGTAACTGACCATGCGCTGGTTCAC	342bp
MYC WT	Forward: CCGCTCGAGTGTAACCTTGCTAAAGGAGTGA<br>Reverse: ACCTTGGGGGCCTTTTCATTGCTAGCTAG	473bp
MYC Mut	Forward: GAGGGTCAAGTTGGTCAGTGTCAGAGTC<br>Reverse: GACTCTGACACTGACCAACTTGACCCTC	473bp
**BGS assay**		
ALKBH5	Forward: GGAAATTTTAGTTAGGGTTTGTT<br>Reverse: CCCACTCTCTAAAAAACTTCAAAAA	290bp
**ChIP assay**		
LKB1	Forward: AAGAAGTGGAAGCGCCAAAG<br>Reverse: GCTTCAAGGGAACGCTCTCA	227bp
LKB1 Negative control	Forward: GGCAGGACTAGGGGGTCAAT<br>Reverse: CAACCCAAGGCAGTGGTGAA	109bp
**MeDIP**		
LKB1	Forward: GGCGGTTCCCTGGTGAAT<br>Reverse: CTTCAAGGGAACGCTCTCAA	178bp
LKB1 Negative control	Forward: GGCAGGACTAGGGGGTCAAT<br>Reverse: CAACCCAAGGCAGTGGTGAA	
**MeRIP**		
SMAD7	Forward: GTCCGAATTGAGCTGTCCGA<br>Reverse: AACCCTCTCTGCCAATGTGT	197bp
SOX2	Forward: GGCAATAGCATGGCGAGC<br>Reverse: TGCTCCAGCCGTTCATGTG	97bp
MYC	Forward: CATCAGCACAACTACGCAGC<br>Reverse: GCTGGTGCATTTTCGGTTGT	120bp
