# Supplementary figures and images for: Suppression of m6A mRNA modification by DNA hypermethylated ALKBH5 aggravates the oncological behavior of KRAS mutation/LKB1 loss lung cancer
Source: Cell Death Dis. 2021 May 20;12(6):518. doi: 10.1038/s41419-021-03793-7 (PMC8137886; doi:10.1038/s41419-021-03793-7)

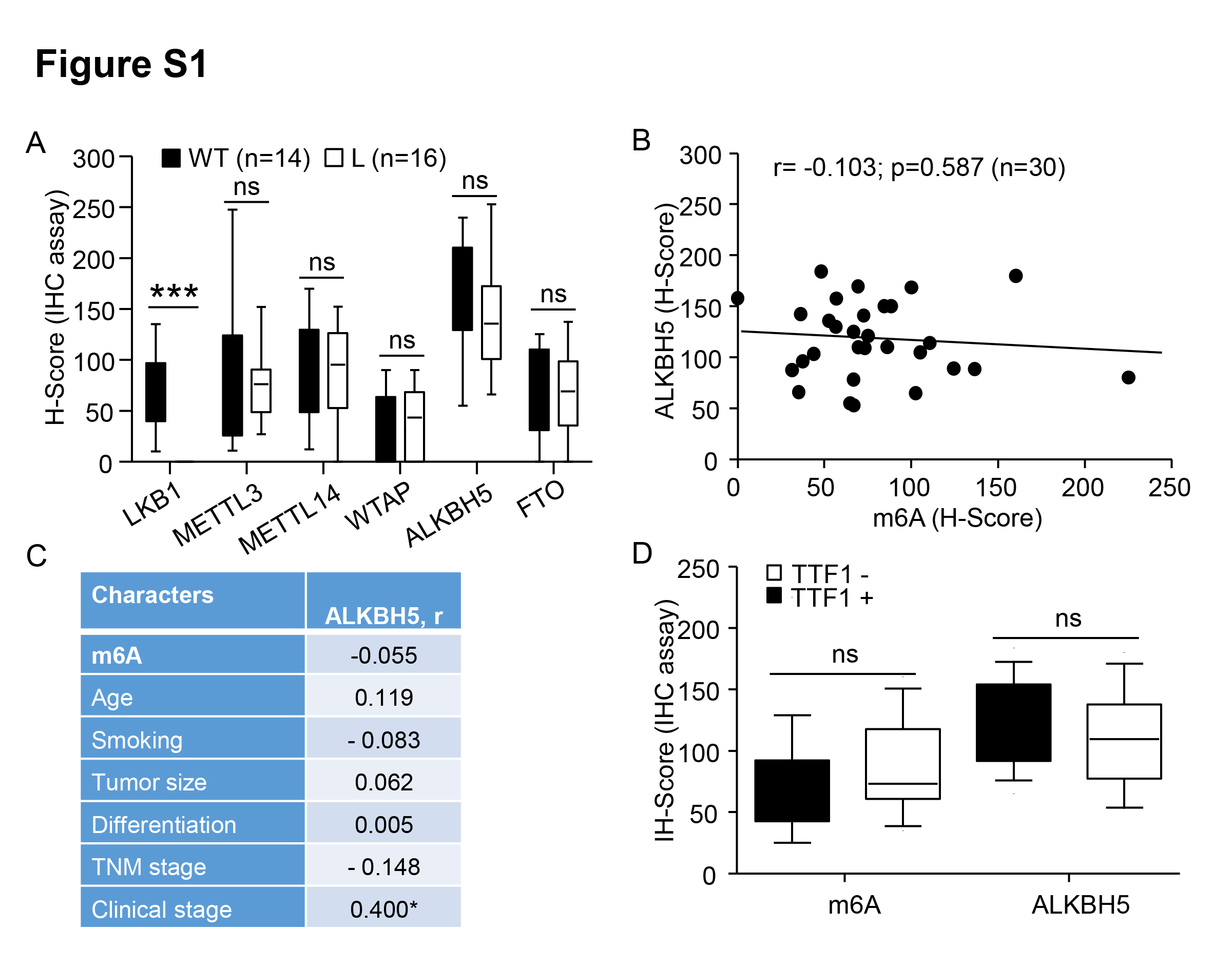

Supplement: Supplementary file 4 — Supplementary figure 1 [file 41419_2021_3793_MOESM4_ESM.tif]

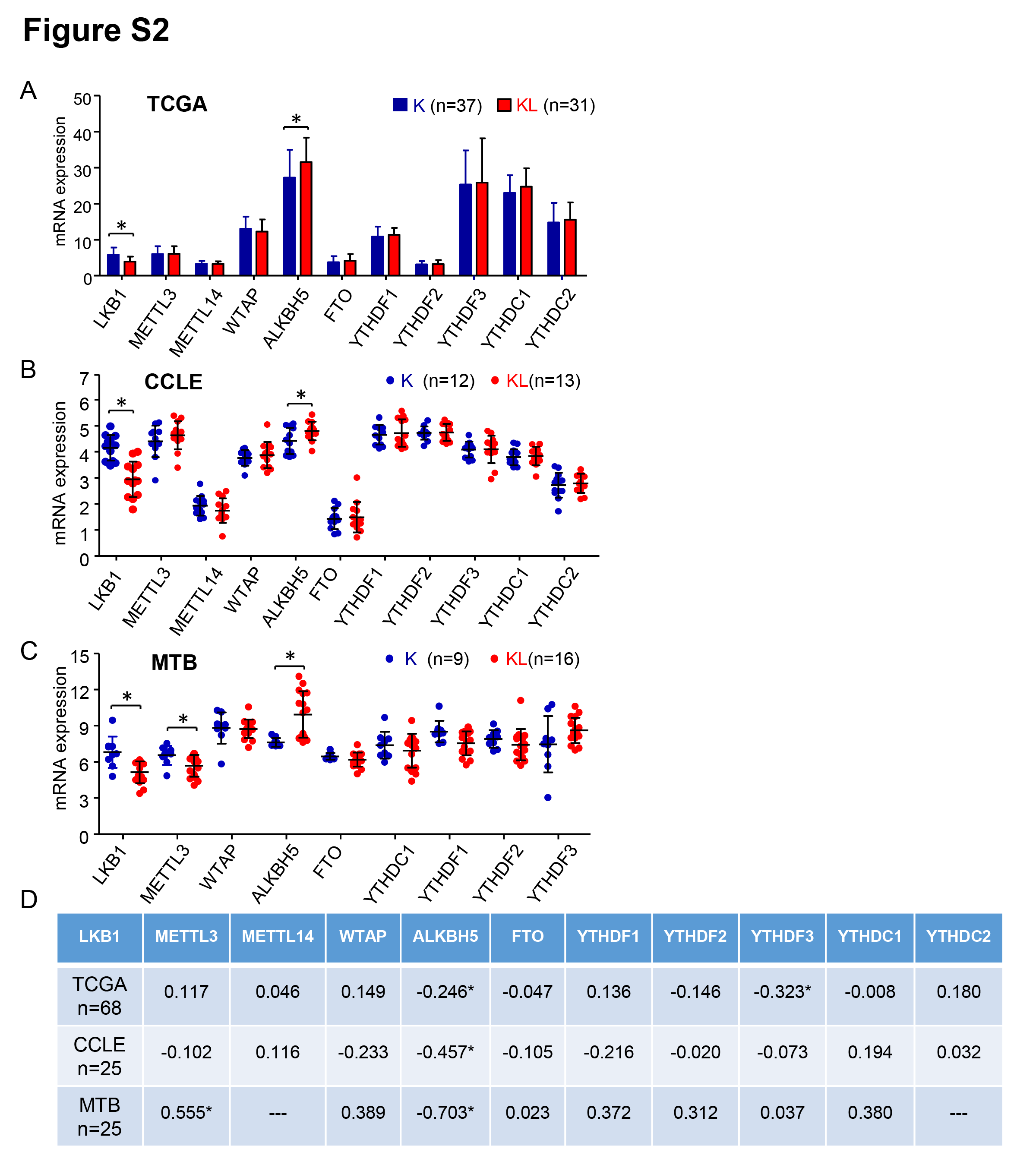

Supplement: Supplementary file 5 — Supplementary figure 2 [file 41419_2021_3793_MOESM5_ESM.tif]

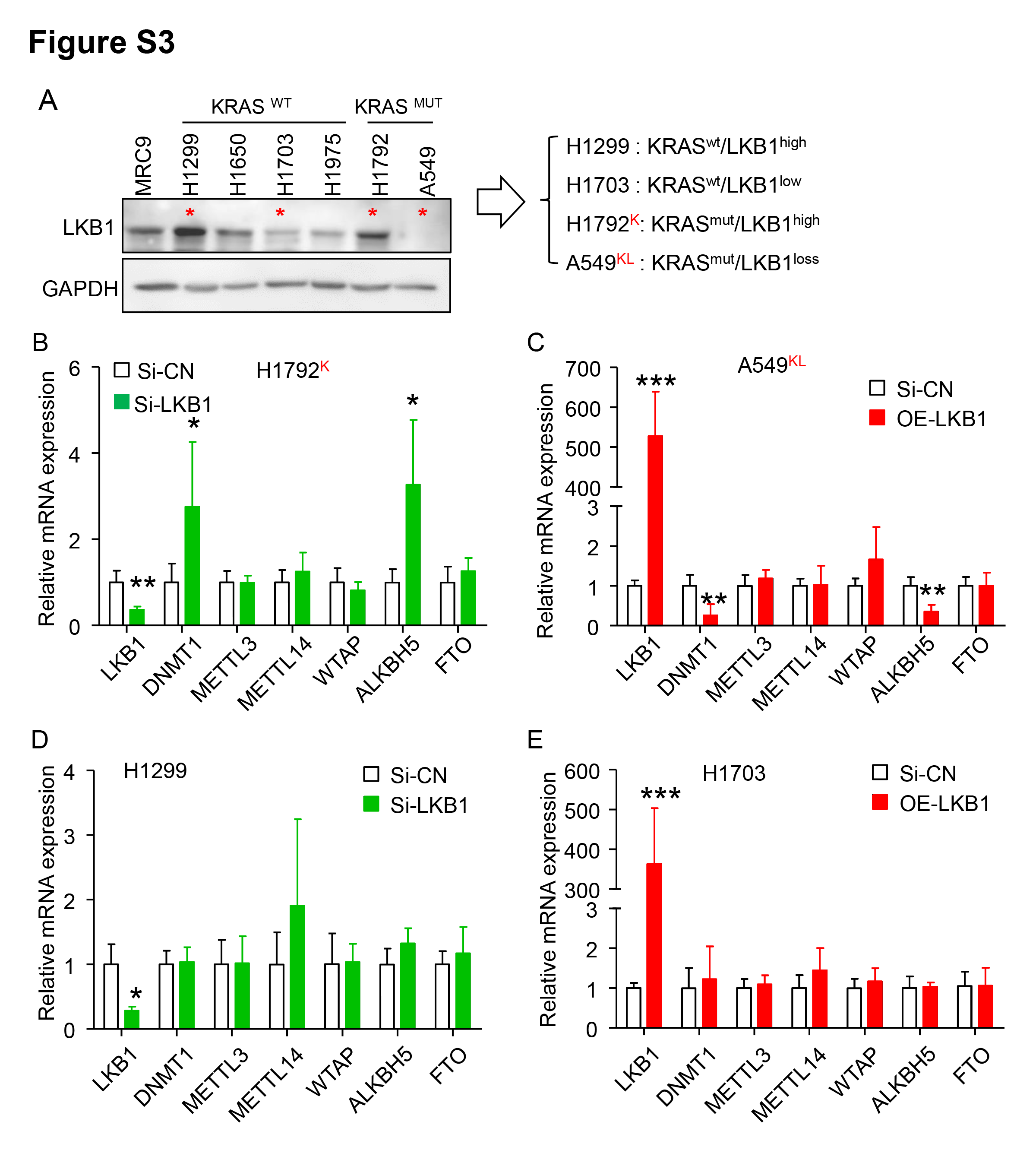

Supplement: Supplementary file 6 — Supplementary figure 3 [file 41419_2021_3793_MOESM6_ESM.tif]

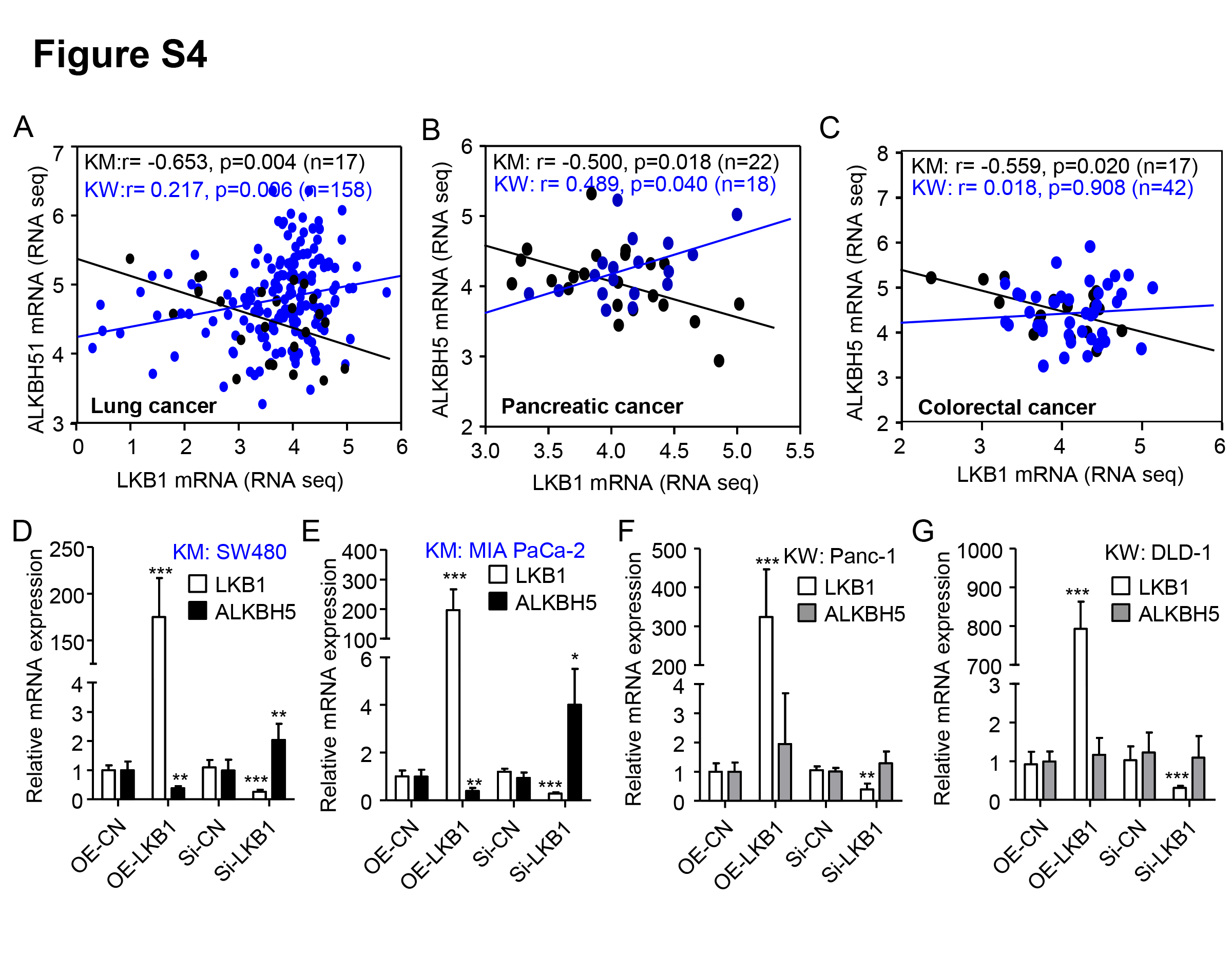

Supplement: Supplementary file 7 — Supplementary figure 4 [file 41419_2021_3793_MOESM7_ESM.tif]

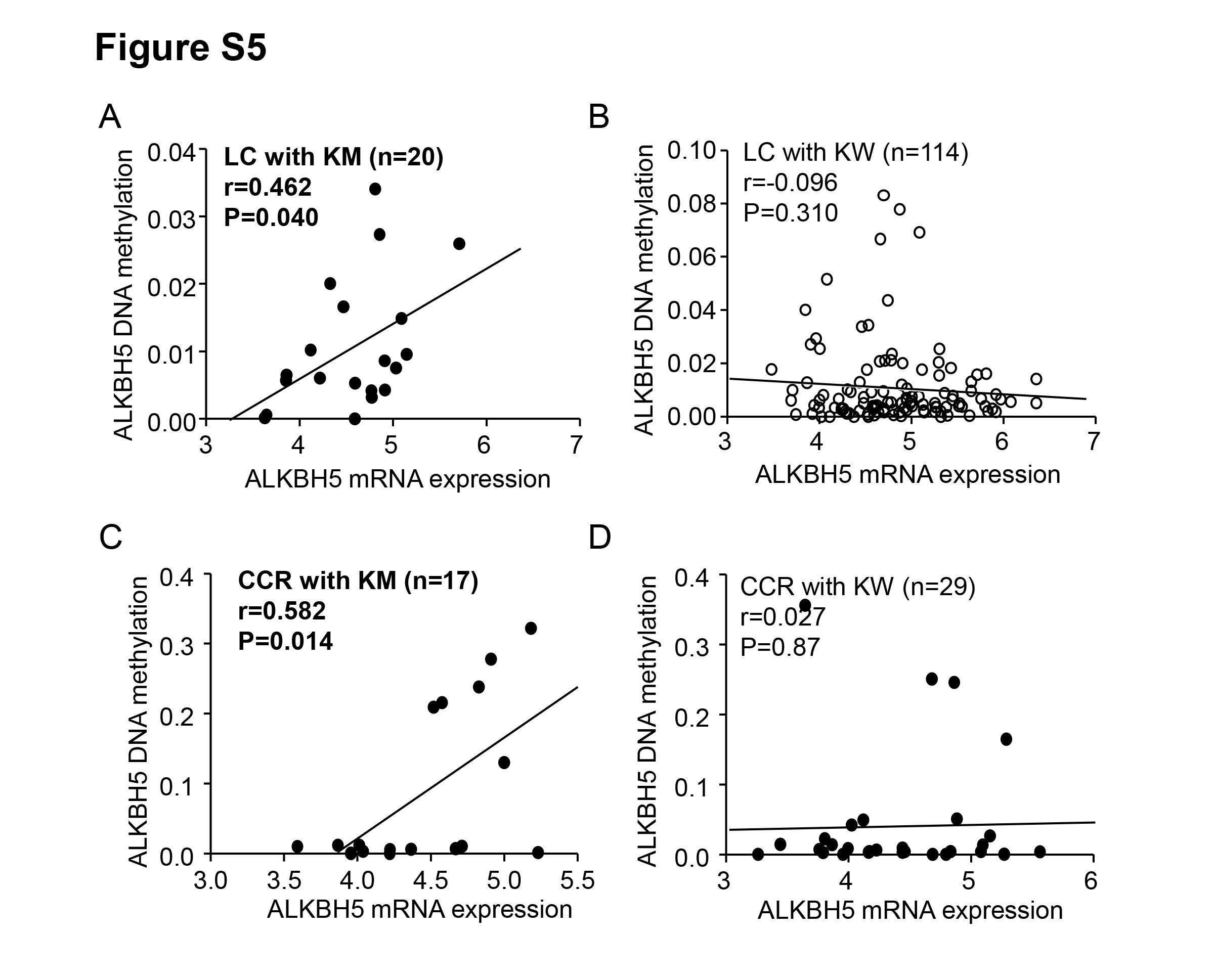

Supplement: Supplementary file 8 — Supplementary figure 5 [file 41419_2021_3793_MOESM8_ESM.tif]

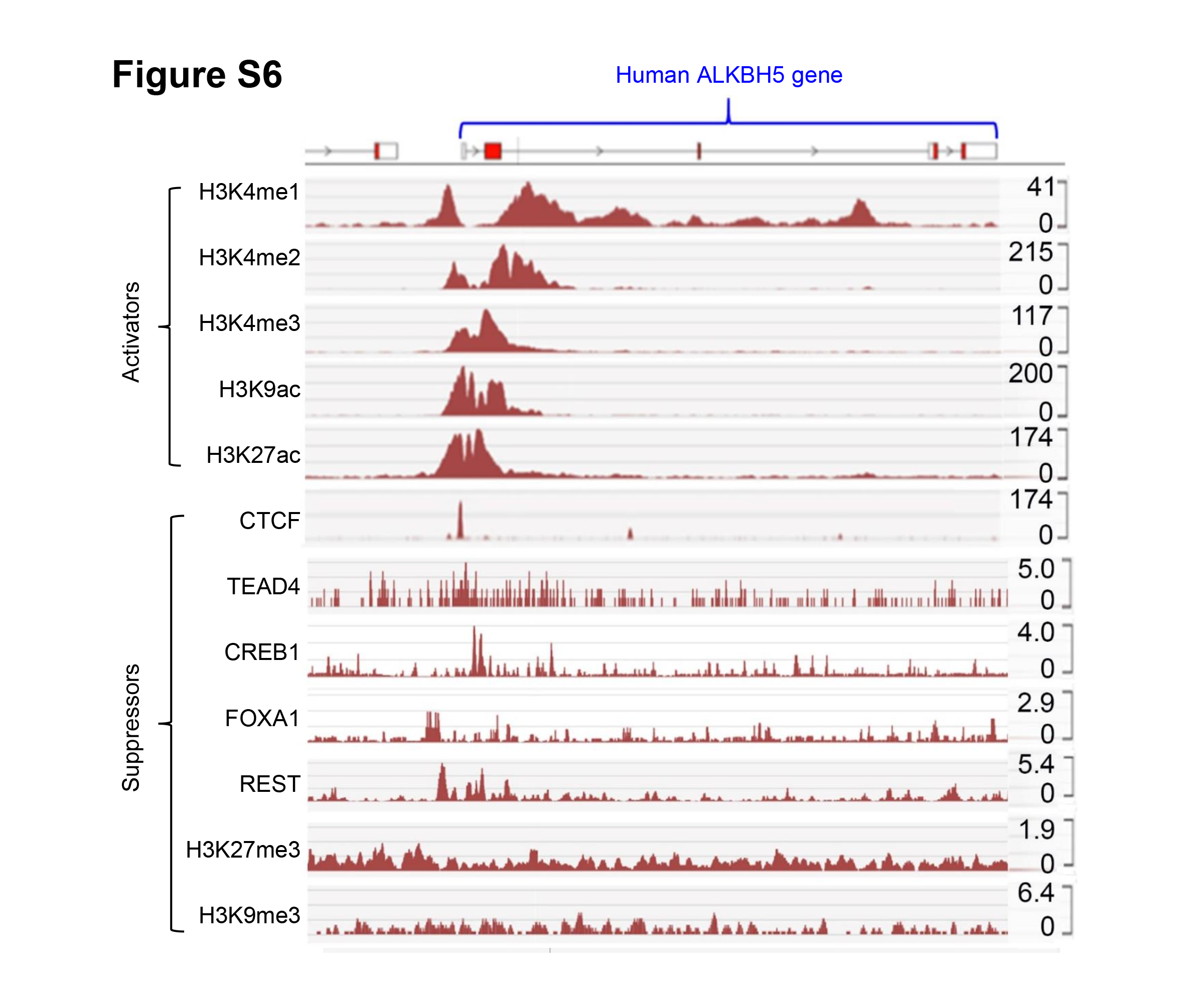

Supplement: Supplementary file 9 — Supplementary figure 6 [file 41419_2021_3793_MOESM9_ESM.tif]

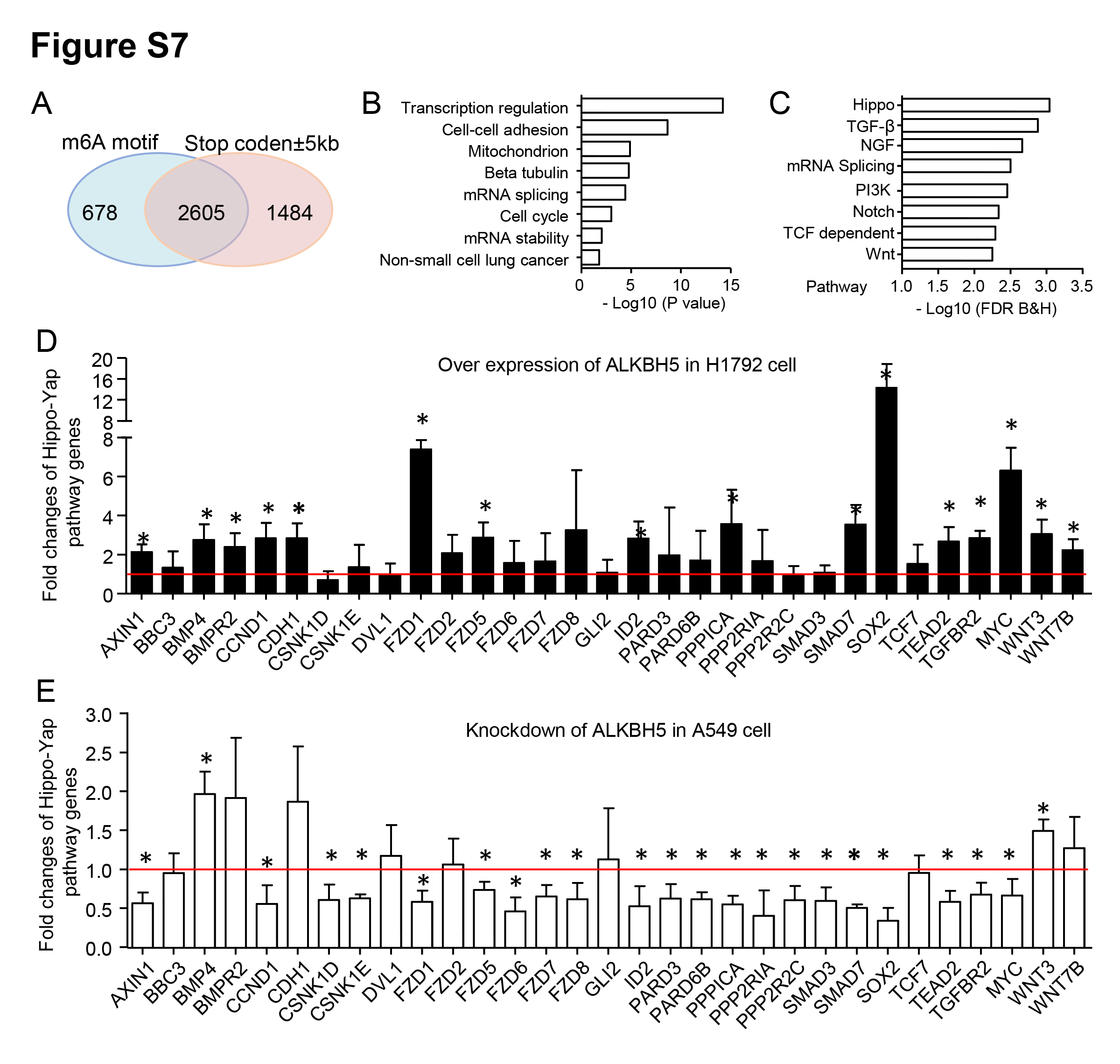

Supplement: Supplementary file 10 — Supplementary figure 7 [file 41419_2021_3793_MOESM10_ESM.tif]

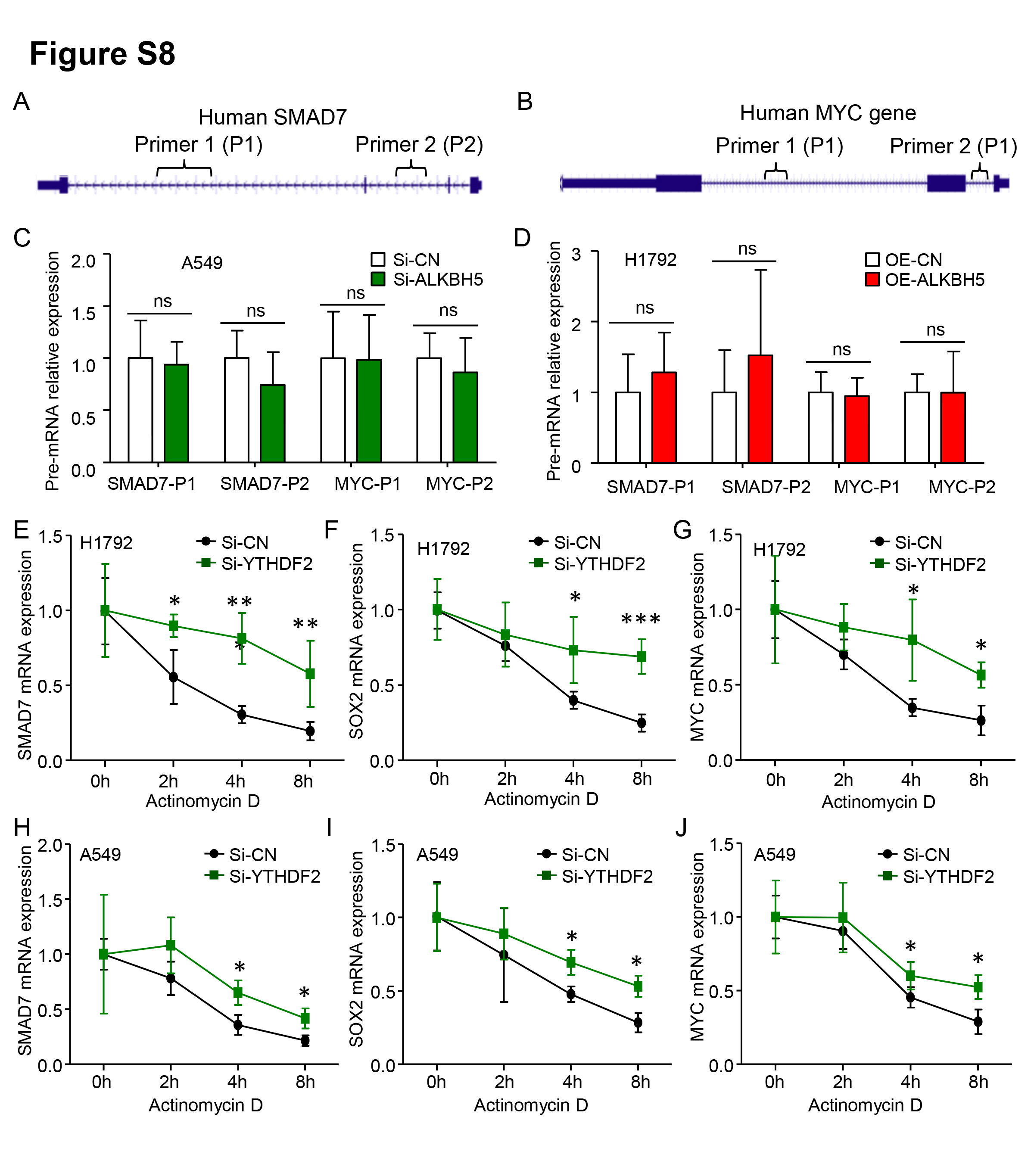

Supplement: Supplementary file 11 — Supplementary figure 8 [file 41419_2021_3793_MOESM11_ESM.tif]

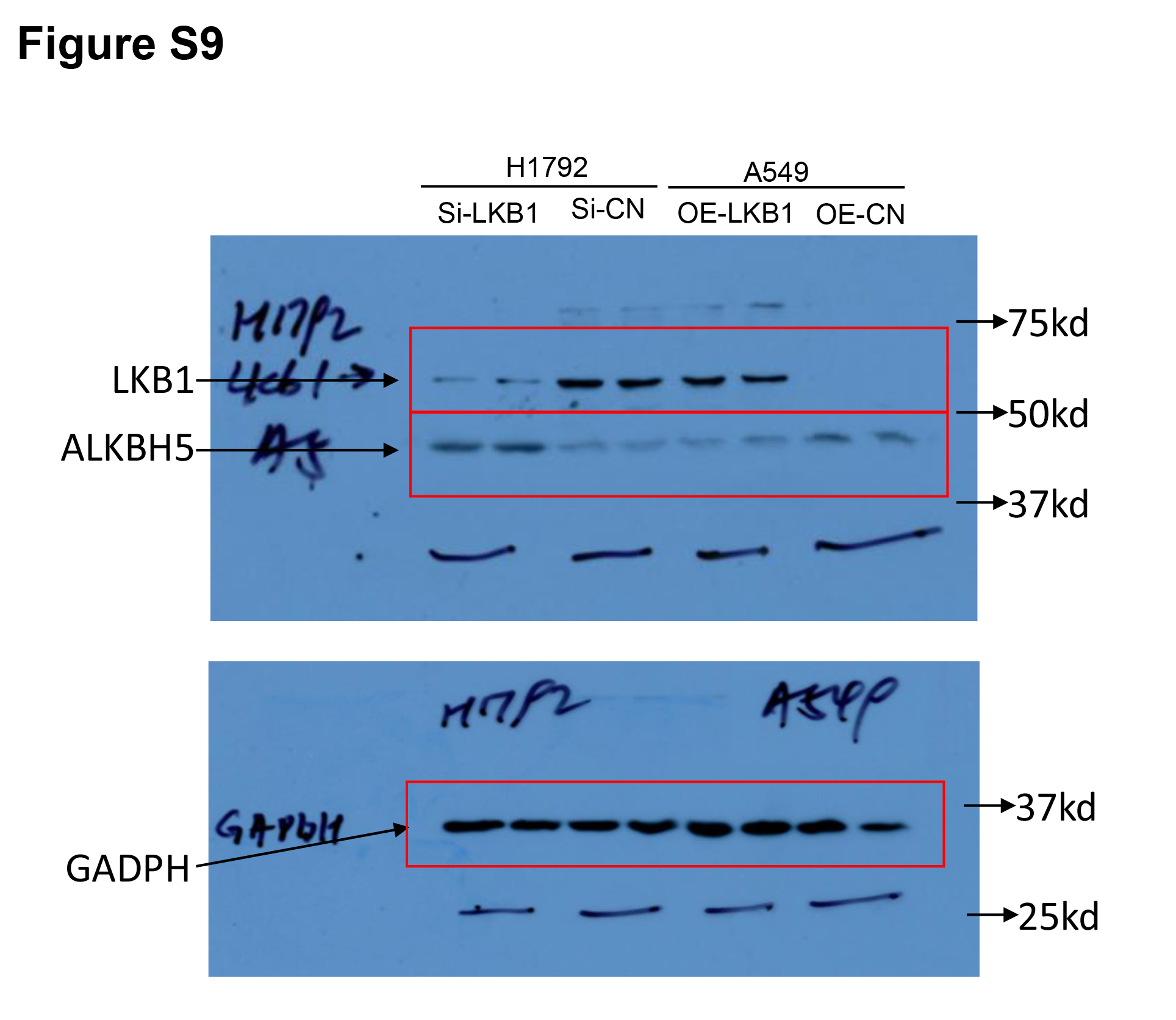

Supplement: Supplementary file 12 — Supplementary figure 9 [file 41419_2021_3793_MOESM12_ESM.tif]

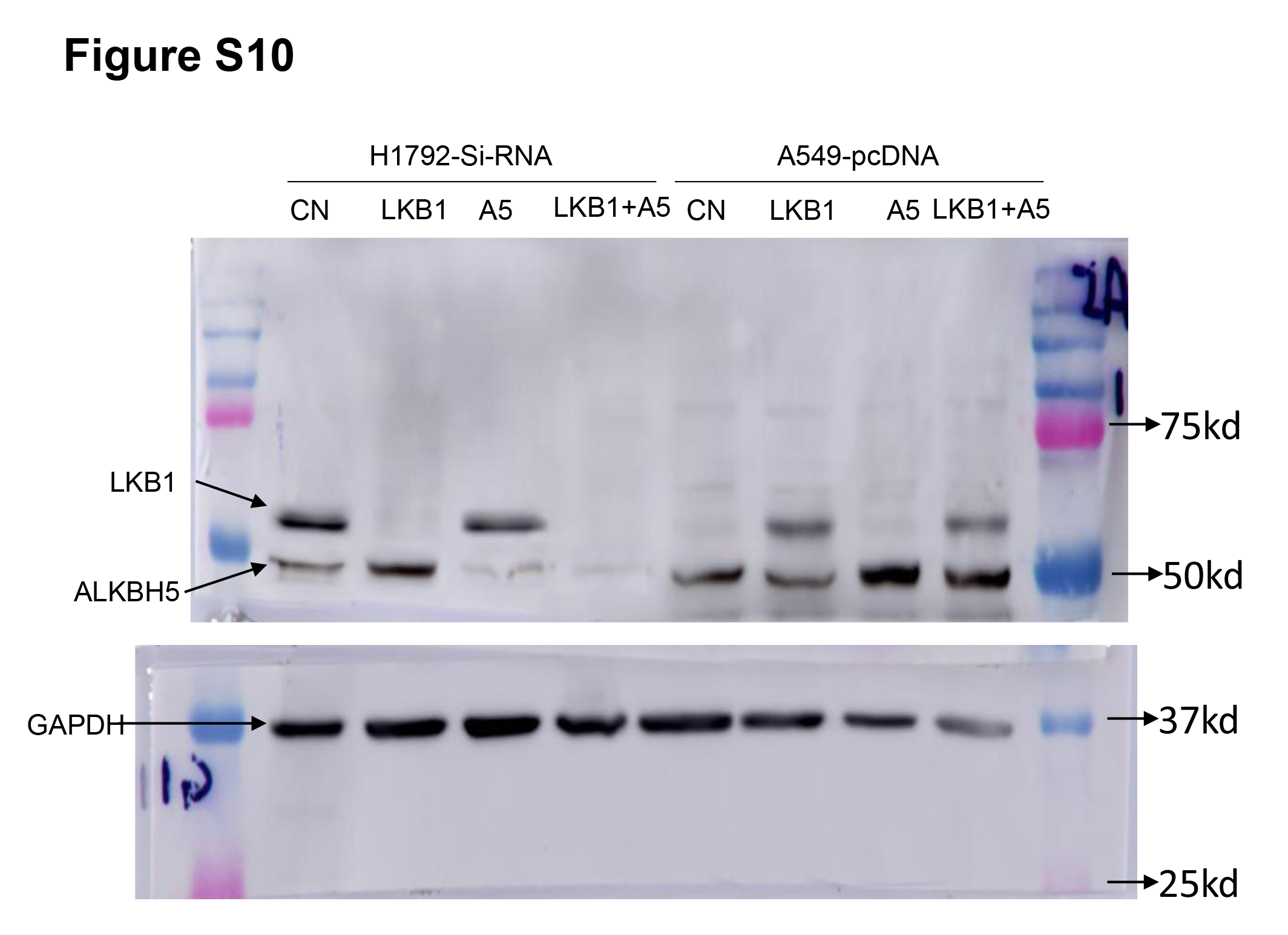

Supplement: Supplementary file 13 — Supplementary figure 10 [file 41419_2021_3793_MOESM13_ESM.tif]

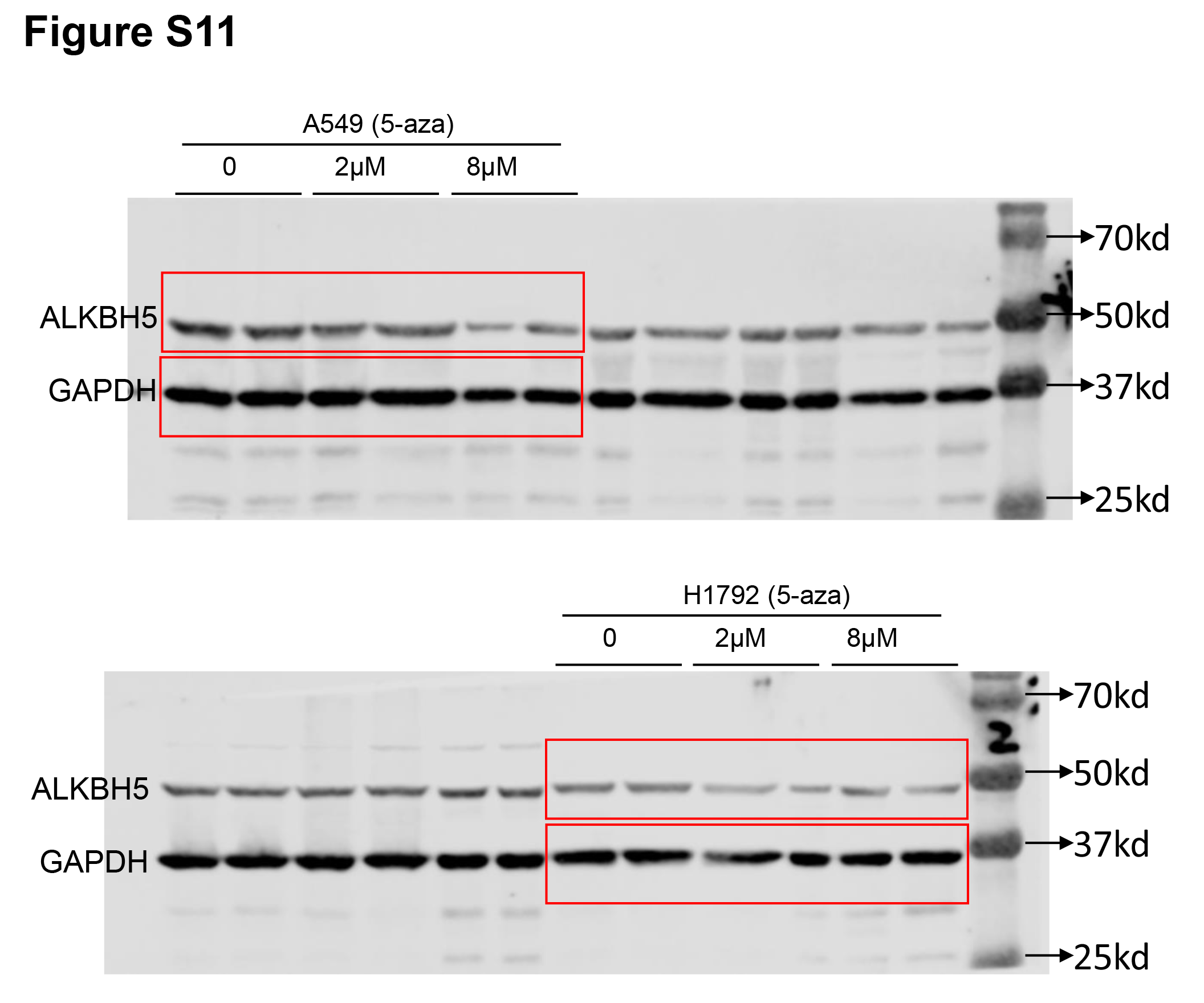

Supplement: Supplementary file 14 — Supplementary figure 11 [file 41419_2021_3793_MOESM14_ESM.tif]

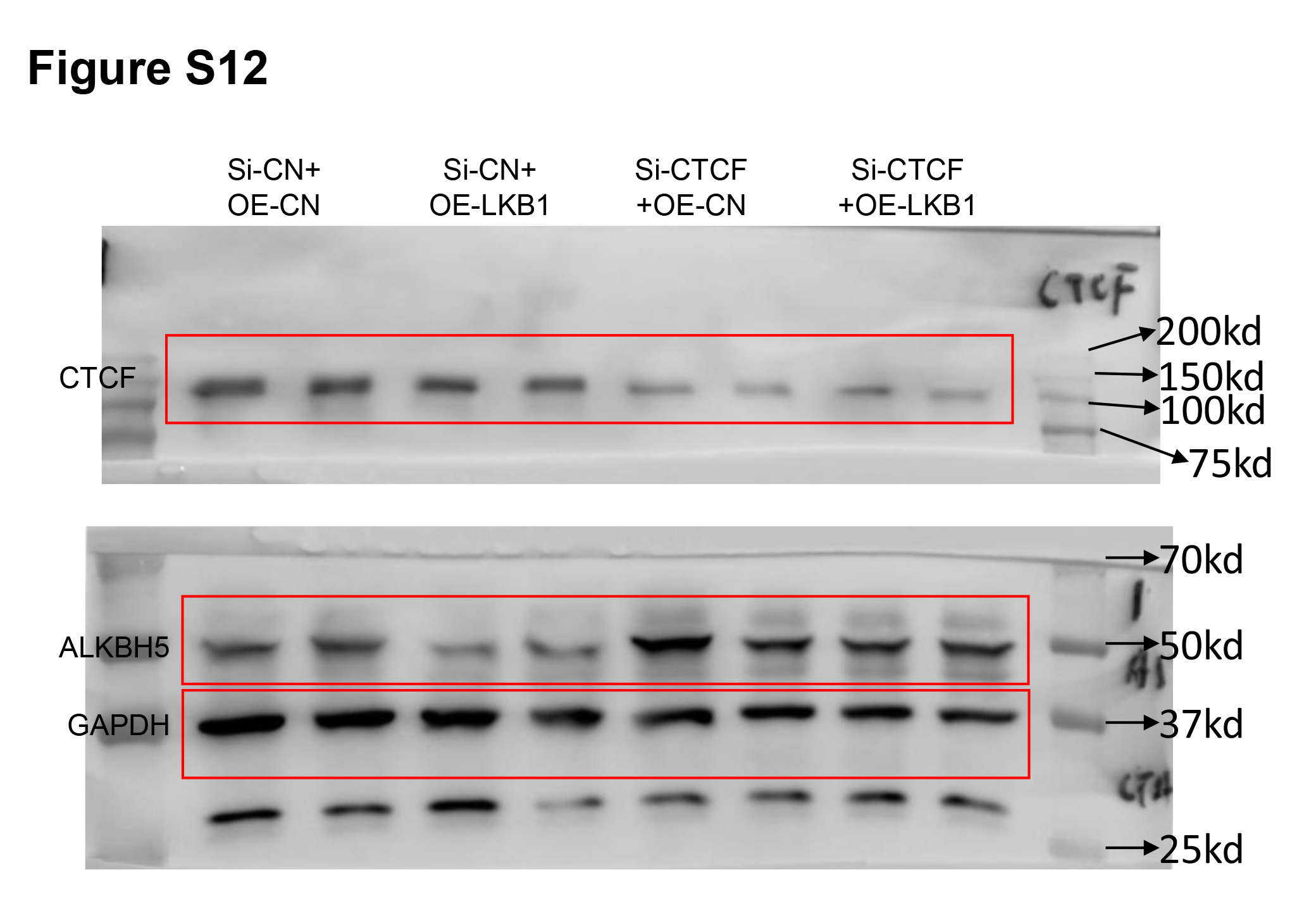

Supplement: Supplementary file 15 — Supplementary figure 12 [file 41419_2021_3793_MOESM15_ESM.tif]

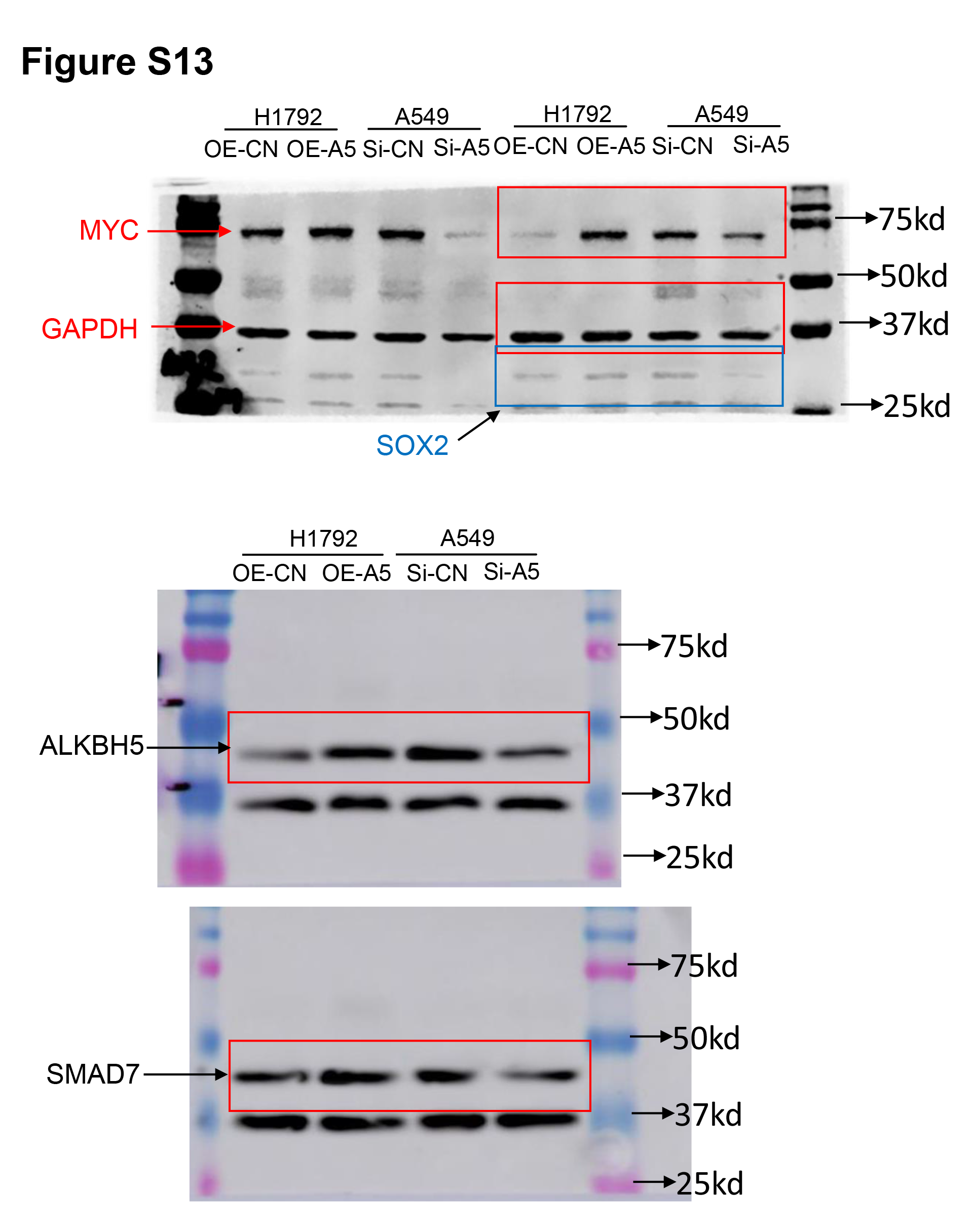

Supplement: Supplementary file 16 — Supplementary figure 13 [file 41419_2021_3793_MOESM16_ESM.tif]

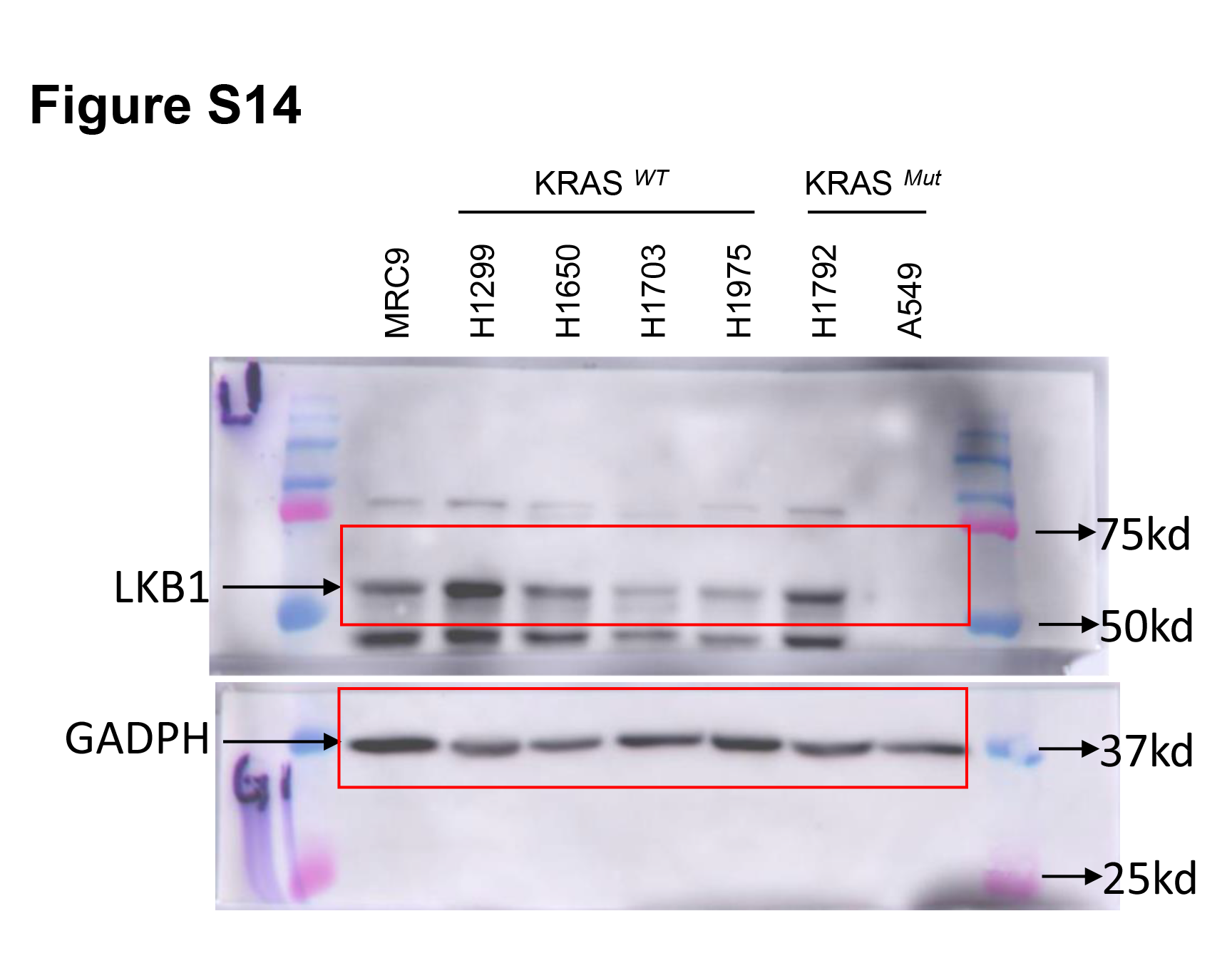

Supplement: Supplementary file 17 — Supplementary figure 14 [file 41419_2021_3793_MOESM17_ESM.tif]
